# Influence of Strongly and Weakly Interfacially Active Asphaltene Particles on Solubility and Crystallizability of Octacosane (C_28_) Model Oils

**DOI:** 10.1021/acs.cgd.5c00801

**Published:** 2025-10-07

**Authors:** Abdulraouf Ali, Ghinwa Yaghy, Alexander Jackson, Chris S. Hodges, Thibaut V. J. Charpentier, Kevin J. Roberts, David Harbottle

**Affiliations:** † School of Chemical and Process Engineering, 4468 University of Leeds, Leeds LS2 9JT, U.K.; ‡ Baker Hughes, 138465 Oilfield Services, Liverpool L33 7TQ, U.K.

## Abstract

Asphaltenes and waxes are two components of crude oil that contribute to flow assurance challenges; however, the interaction between them is rarely studied. This study investigates how asphaltene particles with different physicochemical properties, specifically, remaining asphaltenes (RA) and interfacially active asphaltenes (IAA), influence the crystallization kinetics of C_28_H_58_, a model wax compound. IAA fractions are more polar, primarily due to their higher content of heteroatoms such as sulfur and oxygen, and they tend to form larger particles in solution compared to the RA fraction. At a fixed C_28_-to-asphaltene ratio of 1000:1 (w/w), the introduction of either RA or IAA, especially at low concentrations (10 g/L C_28_ + 0.01 g/L asphaltene), significantly inhibits crystallization, although crystallization continues to proceed via a progressive nucleation mechanism. Inhibited crystallization was evidenced by the increased solubility temperature (*T*_e_), decreased supersolubility temperature (*T*_c,l_), and a broader metastable zone width. These changes are associated with enhanced supersaturation, an elevated free energy barrier to nucleation, increased interfacial tension (γ_eff_), and a higher number of molecules in the critical nucleus (i*). The data supports a 2-step crystallization model: initially, asphaltene particles hinder 3D molecular aggregation (nucleation step), followed by inhibition of crystal growth through adsorption on lateral (*h k 0*) crystal faces. A key finding of this study is the concentration-dependent inhibitory effect of asphaltene particles on wax crystallization. At low concentrations, small asphaltene particles predominate, providing an abundance of asphaltene molecules and nanoaggregates that can fully interact with C_28_ molecules, resulting in stronger inhibition. In contrast, at higher concentrations, larger asphaltene particles become dominant, reducing the number of asphaltene molecules and nanoaggregates in solution. Consequently, their interaction with C_28_ is limited, leading to a weaker inhibitory effect on crystallization. Notably, the inhibitory capacity of IAA is more sensitive to concentration than that of RA. This sensitivity is due to IAA’s strong tendency to self-associate and form large particles, which are less able to interact effectively with C_28_. These findings confirm the significance of asphaltene particle behavior relative to solute concentration in influencing the nucleation pathway and crystallization kinetics of paraffin waxes.

## Graphical abstract

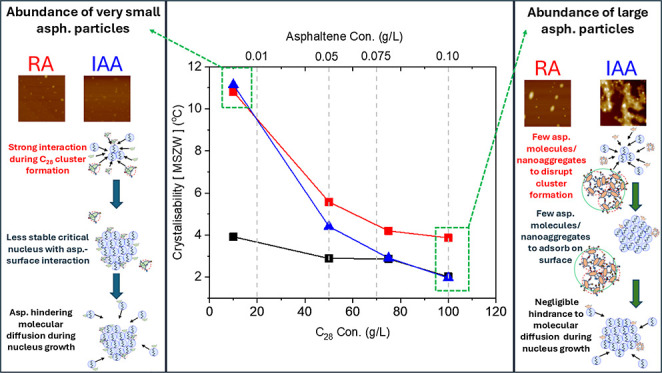

## Introduction

Crude oil is a complex mixture of hydrocarbon compounds that can undergo various degrees of physical transformation during its production, transportation, and refining.^1^ One important aspect of these transformations relates to cold flow problems in transportation which are associated with the crystallization of alkanes (paraffin waxes) when the ambient temperatures drop below the wax appearance temperature (WAT), particularly in deep-water environments. The paraffin wax crystallization can significantly impact hydrocarbon flow leading to blockages which can affect the operational efficiency and pose serious flow assurance issues.^2^ Among the many components present in crude oil, the aggregation behavior of asphaltenes can be considered to be another contributor to flow assurance issues. Unlike waxes, asphaltenes tend to be less sensitive to temperature variation but their behaviors tend to be more governed by their solvency in crude oil. In poor solvents, asphaltenes have a stronger tendency to self-aggregate, forming clusters and particles that can grow quite large (from ∼0.1 μm to several microns). These may deposit on surfaces, restricting flow in pipes and processing equipment.^3–5^ Given that both waxes and asphaltene coexist, within hydrocarbon fuel feedstocks, it is quite surprising that few studies have examined the influence of the presence of asphaltenes upon the crystallizability and associated crystallization kinetics and mechanism of paraffin waxes.

Generally, crystallization processes involve two key stages: nucleation and growth. The kinetics of both stages depend on the magnitude of the supersaturation driving force.^6^ Nucleation involves the three-dimensional formation of the solid crystalline phase from the mother phase, followed by two-dimensional growth on the crystal habit surfaces, leading to the development of macroscopic crystals.^6,7^ Previous literature has mainly focused on the role of asphaltenes on the onset of wax crystallization by determining the wax appearance temperature (WAT), i.e., the temperature at which the first crystal appears upon cooling^1,8–11^ without any examination as to how asphaltenes influence this first nucleation step of the wax crystallization process. Such knowledge is fundamental to gaining a better understanding of the role played by asphaltenes from a first-principles approach as well as the mechanism governing their interaction with the waxy components in crude oils.

The effect of asphaltenes on the wax appearance temperature (WAT) remains inconclusive due to their complex physicochemical nature, with studies reporting both inhibitory and promotive influences. This variation is often attributed to the aggregation state of the asphaltene particles. For instance, Xue et al.^11^ reported a decrease in WAT, suggesting that “dispersed” asphaltene particles hinder wax–wax interactions through steric interference. In contrast, Oh and Deo,^10^ along with Singh et al.^8^ found that “highly aggregated” asphaltenes increased the WAT by serving as nucleation sites that promote wax crystallization. Kriz and Andersen^9^ investigated the effect of asphaltene concentration on WAT and observed that low concentrations (up to 0.01 wt %) led to an increase in WAT, likely due to dispersed particles. However, at higher concentrations (up to 0.5 wt %), the WAT-lowering effect became less evident, which they attributed to increased asphaltene aggregation. In our recent study,^12^ the addition of both highly polar and weakly polar asphaltene subfractions was found to decrease the WAT in model waxy oils. In contrast, Yang and Kilpatrick^13^ and Oliveira et al.^14^ reported no significant effect of asphaltenes on WAT and found no evidence of molecular interactions between waxes and asphaltenes.

Considering the above prospective, it is perhaps important to examine in more detail the addition of asphaltenes to the crystallization of model alkane based fuels,^15^ which has not been studied previously within this context. Analysis of solubility as a function of the asphaltene concentration can provide an indication as to the likely interactions in the solution state, yielding a thermodynamic assessment of its impact upon solution ideality as well as the enthalpies and entropies of dissolution.^16^ The temperature difference between a material’s equilibrium solubility and that at the onset of nucleation, prior to crystal growth, provides a useful measure of the material’s crystallizability or its metastable zone width (MSZW).^7^ The correlation between solution cooling rate and MSZW can be used to assess the relative balance between the kinetic processes of supersaturation generation and the onset of nucleation. Accordingly, a comparative characterization of the MSZW for solutions crystallizing in the absence and presence of asphaltenes provides insight into their influence on nucleation. A widening of the MSZW indicates nucleation inhibition, whereas a narrowing reflects nucleation promotion.^15–19^

Previous studies on the effect of additives on the wax nucleation process have concluded the capability of both small molecules^17,19^ and polymeric-based additives^20,21^ to inhibit and reduce nucleation rate depending on their relative intermolecular binding affinity to solute molecules. Mechanistically, such additives have been suggested to inhibit bulk nucleation through their interaction with solute molecules and through this disturbing the formation of the stable, critically sized solute clusters needed for nucleation. They can also integrate into the bulk structure and/or adsorb onto the surfaces of growing crystals, hence disturbing surface nucleation by restricting the ability of further solute molecules to incorporate into the crystal structure.^19,22^ For instance, Kaskiewicz et al.^21^ showed a reduction in both the solubility and crystallizability of eicosane (C_20_) when adding a commercially available cold flow improver additive. Their data suggested a change in the solution thermodynamics induced by intermolecular interactions (dominated by van der Waals (vdW) interactions) between C_20_ and the additive molecules, leading to the formation of less stable eicosane clusters, thus inhibiting nucleation.

In those studies, the additives are fully solubilized, allowing intermolecular interactions between solute and additive molecules to directly influence the nucleation process. By contrast, asphaltenes are not necessarily solubilized; instead, they tend to exhibit a size hierarchy, as described by the Yen-Mullins model, where they readily self-associate into nanoaggregates, clusters, and particles,^4,23^ depending on their concentration and the solvent polarity.^5,24,25^ Consequently, the influence of asphaltenes on wax nucleation and the underlying mechanistic processes of inhibition or promotion is likely to be complex, providing strong motivation for further investigation.

The type of solvent can significantly affect both the solubility and crystallizability of alkanes.^18,26–28^ A study on the solubility of C_18_ and C_32_ in a diverse range of solvents, including ethanol, heptane, triglycerides, and water, showed a pronounced solvent effect.^26^ In highly polar solvents, strong solute self-association was observed, resulting in poor solubility because the solution structure favored solvent–solvent interactions over solute solvation. This was confirmed by Kaskiewicz et al.^18^ who found that the solubility of C_20_ was the highest in essentially apolar toluene solvent which decreased with increasing content of the more polar acetone. Jackson et al.^28^ highlighted the dependence of solvent effect on the length of alkane chains, revealing a reduction in the solubility and crystallizability of C_16_ to C_19_ alkanes in toluene when compared to dodecane solvents. Hence, these studies suggest the added value of investigating the effect of solvent as a part of an investigation of the role of asphaltenes on wax crystallization.

This study develops an understanding on the effect of asphaltene subfractions^5,29^ on the solubility and crystallizability of a model waxy system. Asphaltene subfractions are separated from whole asphaltenes (WA) into strongly and weakly interfacially active asphaltenes by partitioning at an oil–water interface.^4,23,30^ These two subfractions of WA are commonly termed interfacially active asphaltenes (IAA) and remaining asphaltenes (RA),^31^ and have been shown to exhibit contrasting physicochemical properties.^5,29^ The IAA fraction is more polar, which is attributed to the higher heteroatom content, specifically oxygen, as well as being more multicore and aliphatic compared to the RA fraction.^5,23^ The IAA fraction also has a greater tendency to aggregate even in good solvents and tends to form nanoaggregates that are larger and more porous than those formed by RA. It is worth noting that the IAA fraction is ∼2 wt % of WA, and while it can be considered to be a minor fraction, it has been shown to impact upon issues related to flow assurance, such as deposition and emulsion stablisation.^30,32^

The work presented here encompasses polythermal crystallization and dissolution onset characterization of octacosane (C_28_H_58_, hereinafter referred to as C_28_) acting as a representative wax in fuel solutions which has been characterized with and without the presence of RA and IAA. The selection of C_28_ was based on the fact that it is representative of the median chain length typically found in paraffinic crude oils and fuels.^33^ The crystallization and dissolution temperatures were determined through turbidity measurements and the fundamental mechanisms and nucleation kinetics by which nucleation occurs within the solutions were assessed using the Kashchiev-Borissova-Hammond-Roberts (KBHR) method.^18,27,34^

## Materials and Methods

C_28_ was purchased from Alfa Aesar (melting point of 61–63 °C and >99% purity), dodecane was supplied by Sigma-Aldrich (purity of 96% and boiling point of 215 °C) and toluene was supplied by Fisher Scientific (purity of ≥99.8% and boiling point of 111 °C). These materials were used as received, without further purification Asphaltenes were extracted from a Colombian (Llanos region) heavy crude oil with an API of 13.6 (at 60 °F).

### Asphaltene Extraction and Subfractionation

Whole asphaltenes (WA) were extracted by mixing heavy crude oil (asphaltene content = 39.5 wt %)^5^ with *n*-pentane at a ratio of 40:1 (v/v) for 10 min at 15,000 rpm (T18 Ultra-TUR RAX, IKA, U.K.). The solution was then left undisturbed for 24 h before filtering (8 μm Whatman #2 filter paper) under gentle vacuum. To avoid residual contamination of the asphaltenes by other species, the filter cake was repeatability washed with pentane until the filtrate ran clear. The washed asphaltenes were then dispersed in toluene (20:1 solvent to asphaltenes mass ratio) and centrifuged (Heraeus Megafuge 16 Centrifuge, ThermoFisher Scientific, U.K.) at 10,000 rpm for 40 min to remove any fine mineral solids. The supernatant was removed using a wide-bore pipet and the toluene evaporated to leave the WA sample.

The WA sample was subfractionated into remaining asphaltenes (RA) and interfacially active asphaltenes (IAA) following the method of Ballard et al.^5^ and Yang et al.^31^ The fractionation method separates asphaltenes based on their interfacial activity at an oil–water interface. In brief, 10 mL of deionized water was homogenized with 100 mL of 10 g/L WA in toluene for 15 min at 24,000 rpm. The stable emulsion was left for 12 h before separating the supernatant from the emulsion-bed. The supernatant was then left in a fume hood to evaporate the solvent to recover the RA fraction. The emulsion-bed was gently washed with excess toluene to remove any loosely bound asphaltenes from the water–oil interface, before drying the emulsion in a vacuum oven at 60 °C for 12 h to recover the IAA fraction. From 10 g WA, the amount of IAA extracted was between 0.15 and 0.2 g, meaning the quantity of IAA in WA is ∼2%. The chemical and composition analysis of the fractionated RA and IAA are shown in Figure S1 and Table S1.

### Solution Preparation

The pure octacosane solutions in 6:4 dodecane/toluene (hereinafter referred to as 6:4 DodecTol) and toluene were prepared at four solute concentrations: 10, 50, 75, and 100 g/L of C_28_. To prepare solutions containing RA or IAA at 1000:1 wt/wt C_28_: asphaltene ratio, 0.01, 0.05, 0.075, and 0.1 g/L asphaltenes were added, respectively. For 6:4 DodecTol, a 10 mL solution was prepared by adding the desired amount of C_28_ powder (depending on the solute concentration) into a 15 mL glass vial, using a weighing scale (Fisher Scientific) with ±0.1 mg accuracy. Six mL of dodecane was pipetted (Fisherbrand micropipette) into the vial and then heated at 80 °C for 5 min while gently stirring using a magnetic stirrer. Meanwhile, the desired amount of RA or IAA was weighed into a 10 mL vial, and 4 mL of toluene was subsequently added and then sonicated at 80 °C for 5 min. The asphaltene solution was then poured into the C_28_ solution at 80 °C and stirred for a further 5 min. Finally, 1 mL of the final solution was pipetted into 1.5 mL glass vials sealed with septum and a standard 7 × 2 mm magnetic stirrer was added to each vial. Four vials were made for each sample concentration. A schematic flowchart of the sample preparation procedure is shown in Figure S2. The same protocol was followed with toluene solutions. It is important to note that pure dodecane was not included in this study as the asphaltene subfractions are completely insoluble in purely aliphatic solvents, making it impossible to assess their effect on octacosane crystallization kinetics.

### Polythermal Measurements

Technobis Crystal 16 crystallization system was used to conduct crystallization/dissolution experiments. The purpose of these experiments is to determine the equilibrium crystallization temperature at the kinetic limit (*T*_c,l_) and the equilibrium dissolution temperature (*T*_e_), which were used to assess the C_28_ solubility using van’t Hoff analysis as well as the nuceltion kinetic parameters in the KBHR model to assess the effective interfacial tension (γ_eff_), the critical nucleus radius (r*), and the number of molecules in the critical nucleus (i*). The procedure of data analysis are described in the following sections. For each solution concentration, 4 × 1.5 mL glass vails were loaded into the thermally controlled block of cells. The sample cells were continuously agitated by stirring at 700 rpm, heated to 40 °C, and held for 15 min to ensure complete dissolution of C_28_. Then the temperature was cooled to −10 °C at a specified rate and held for 15 min before being heated to 40 °C at the same rate. Consecutive cooling and heating cycles were run at different rates of 0.2, 0.5, 1, 2, and 3 °C/min. All measurements were repeated at least three times with the mean and standard deviation values for both the crystallization and dissolution temperatures reported.

The temperatures at which crystallization (*T*_c_) and dissolution (*T*_diss_) occur were determined by analyzing changes in the turbidity profile, which measures the amount of light transmitted through the solution as a function of temperature. *T*_c_ was taken as the onset point where the light transmittance decreased to ∼95%, while *T*_diss_ was taken as the point where the light transmittance was ∼100% for all measurements. For a valid data comparison, samples with the same C_28_ concentrations (e.g., 100 g/L) with and without asphaltenes were tested on the same block to reduce for the measurement variability and standard deviation. In addition, the accuracy of the temperature measurements obtained from the block were calibrated against an independently measured temperatures using a digital thermometer. The calibration curve for the Crystal 16 unit used in this study is shown in Figure S3.

### Differential Scanning Calorimetry

The Mettler Toledo DSC-1 STARe system was used to measure the enthalpy of fusion (Δ*H*_fus_) and melting temperature (*T*_m_) of C_28_. Samples of ∼13 mg were loaded into aluminum standard pans with a capacity of 40 μL (Mettler Toledo). The experimental procedure involved subjecting the sample to a temperature profile, starting with heating it to 80 °C and maintaining this temperature for 15 min to ensure complete dissolution. Subsequently, the sample was cooled to −30 °C and held at this temperature for 15 min to allow for equilibration. Throughout both the heating and cooling cycles, a constant rate (*q*) of 5 °C/min was applied. The temperature cycle was repeated 3 times to obtain reliable mean values. Δ*H*_fus_ was determined by integrating the endothermic peaks and *T*_m_ was taken at the peak of the monoclinic structure using STARe Evaluation Software (Mettler Toledo). The determined melting point and the enthalpy of fusion are shown in Table S2 and Figure S4.

### Atomic Force Microscopy

The polythermal experiments on Crystal 16 were conducted for relatively long-time spans (up to 24 h at 0.2 °C/min) while being continuously agitated (homogenized) at 700 rpm. The agitation leads to a wide range of asphaltene particle sizes present in the solution (Figure S5) ranging from submicron to several microns. Therefore, it was crucial to capture the differences in aggregation behavior of RA and IAA as a function of concentration during the polythermal experiments, so the crystallization kinetics of C_28_ are better understood.

AFM imaging provided an estimate of RA or IAA particle sizes that were aged and continuously agitated, replicating Crystal 16 test conditions. Following the method of Balestrin et al.,^35^ AFM sample substrates were made by sticking freshly cleaved mica on a glass slide and then pipetting 1 to 2 droplets of RA or IAA solutions on the mica sheet. Subsequently, the glass slide was placed on a preheated hot stage at 80 °C to allow fast solvent drying and eliminate further asphaltene aggregation due to aging. In this experiment, 0.1 and 0.01 g/L RA or IAA in 6:4 DodecTol or toluene solutions were used to represent the upper and lower bounds of the concentrations studied in this work. The samples were prepared as previously described but with no addition of C_28_.

The AFM images of RA and IAA-coated mica sheets were obtained using an Innova AFM (Bruker, USA) operated under tapping mode using silicon nitrate cantilevers (model PPP-NCHR, nanosensors, resonance frequency 330 kHz, spring constant 42 N m^–1^). Images were recorded at a scan rate of 0.5 Hz with a resolution of 512 lines/image and 512 points/line at a room temperature of 21 °C. Images were acquired with the amplitude set point at 98% of the free amplitude before engaging the tip. Different locations were imaged for all mica substrates to ensure representative observations and avoid the solvent drying line.

### Data Analysis—Solubility and MSZW

For the four samples of the same solute-asphaltene concentration, the average values of *T*_c_ (crystallization temperature) and *T*_diss_ (dissolution temperature) were derived based on three repeated cooling/heating cycles. These values were then plotted against the rate at which the solution was cooled to determine the equilibrium temperatures associated with crystallization (*T*_c,l_) and dissolution (*T*_e_) by extrapolating them to a cooling rate of 0 °C/min. All plots of *T*_c_ and *T*_diss_ as a function of *q* used in the study are shown in Figures S7 and S8. By plotting the extrapolated values against the concentration of the solute, solubility and supersolubility curves were obtained for each solution system. The solubility represents the equilibrium dissolution temperature (*T*_e_), whereas the supersolubility represents the limit of metastability for a system, as determined from the extrapolation of the crystallization temperature with cooling rate (*T*_c,l_). An example of the determined solubility and supersolubility curves are shown in Figure 2a. The metastable zone width (MSZW) values were taken as the difference between the *T*_e_ and *T*_c_, _l_. The level of supersaturation at equilibrium can be determined from the ratio of the absolute solute concentration at the equilibrium supersolubility (*c*) and that of solubility (c*). This is known as the supersaturation ratio which reflects the free energy barrier that must be overcome for nucleation to occur (see eq 1).

1 $$S=\frac{c}{c^{*}}$$

The calculated *T*_e_ data were used to perform the van’t Hoff analysis. The aim of this analysis is to determine how the studied systems deviate from ideality and to provide insight into the interactions between solute and solvent molecules. The mole fraction of the solute (*x*) can be related to that of the ideal state (*x*_ideal_) through its activity coefficient (γ) (see eq 2). This relationship is derived by equating the activities of the ideal and nonideal states, based on the standard definition of activity.

2 $$\gamma =\frac{x_{ideal}}{x_{e}}$$

The solution behaves ideally when γ = 1, whereas γ > 1 and γ < 1 describes the solution being less and more than ideal, respectively. The attraction forces between like molecules (solute–solute or solvent–solvent) would be preferred over those of unlike molecules for γ > 1, and vice versa for γ < 1.

The ideal solid–liquid equilibrium can be expressed by van’t Hoff’s equation with the assumption of negligible contribution of heat capacity (*C*_p_) over the temperature range considered as follows^7,36^

3 $$\ln x_{ideal}=-\frac{{\Delta H}_{fus}}{R}\left[\frac{1}{T}-\frac{1}{T_{m}}\right]$$

where, *x*_ideal_ is the solute mole fraction, *T* is the solution temperature (K), *T*_m_ is the melting temperature of pure solute (K), *R* is the gas constant (8.314 J/mol. K), and Δ*H*_fus_ is the molar enthalpy of fusion of the solute (J/mol). In this study, *T*_m_ and Δ*H*_fus_ of C_28_ were determined from the DSC data shown in the Supporting Information.

At the melting point (*T*_m_), the enthalpy of fusion (Δ*H*_fus_) can be related to the entropy of fusion (Δ*S*_fus_) by eq 4

4 $${\Delta S}_{fus}=\frac{{\Delta H}_{fus}}{T_{m}}$$

Thus, eq 4 can be rewritten as

5 $$\ln\left(x\right)=-\frac{{\Delta H}_{fus}}{RT}+\frac{{\Delta S}_{fus}}{R}$$

In a nonideal solution, the deviation from ideality can be accounted for by modification of the right-hand side of the van’t Hoff expression, allowing the solubility to be written as

6 $$\ln\left(x\right)=-\frac{{\Delta H}_{diss}}{RT}+\frac{{\Delta S}_{diss}}{R}$$

where, *x* is the mole fraction of the solute at saturation, and Δ*H*_diss_ and Δ*S*_diss_ are the molar enthalpy and entropy of dissolution of the solute, respectively. Using this expression, by plotting ln­(*x*) versus 1/T for ideal or experimental data, Δ*H*_diss_ and Δ*S*_diss_ can be obtained from the slope ( $-\frac{{\Delta H}_{diss}}{R}$ ) and intercept ( $\frac{{\Delta S}_{diss}}{R}$ ), respectively.

The enthalpy (Δ*H*_mix_) and entropy (Δ*S*_mix_) of mixing were calculated from the deviation in Δ*H*_diss_ and Δ*S*_diss_ from ideality using the following equations

7 $$\Delta H_{mix}=\Delta H_{diss}-\Delta H_{diss}^{ideal}$$

8 $$\Delta S_{mix}=\Delta S_{diss}-\Delta S_{diss}^{ideal}$$

### Data Analysis—Nucleation Mechanism and Kinetics

The Kashchiev-Borissova-Hammond-Roberts (KBHR)^37,38^ method was utilized to determine the nucleation mechanism and associated kinetics parameters for each system. The method relates the relative critical undercooling (*u*_c_) to the cooling rate (*q*).

9 $$u_{c}=\frac{\left(T_{e}-T_{c,l}\right)}{T_{e}}$$

The slopes from a fitted linear regression of ln *u*_c_ vs ln *q* provides information as to the dominant nucleation mechanism. A slope >3 indicates progressive nucleation (PN), whereas a slope <3 is instantaneous nucleation (IN). During the IN process, all crystal nuclei in solution emerge simultaneously and subsequently grow to form crystals. Whereas, in PN new crystal nuclei are continuously formed in the presence of already forming crystals.

For the PN case, the *u*_c_ (*q*) dependence can be related through the number of crystallites at the detection point (*N*_det_), which is described through the parameter *q*_0_ in eq 10.

10 $$\ln q=\ln\left(q_{0}\right)+3\ln u_{c}-\frac{b}{\left(1-u_{c}\right)u_{c}^{2}}$$

where

11 $$q_{0}=\frac{VK_{J}T_{e}}{N_{\det}2b}$$

where *V* is the volume of the solution, *K*_J_ is the nucleation rate constant.

Fitting ln *q* vs *u*_c_ enabled *q*_0_, and *b* parameters to be determined for the calculation of the nucleation kinetic parameters being the nucleus effective interfacial tension γ_eff_, the critical nucleus radius (r*)­and the number of molecules in the critical nucleus (i*), through eqs 12–14.

12 $$b=\frac{k_{n}v_{0}^{2}\gamma_{eff}^{3}}{{kT}_{e}\lambda^{2}}$$

where *k*_n_ is the nuclei numerical shape factor (i.e., 4π/3 for spheres^39^), *v*_0_ is the volume occupied by a solute molecule in the crystal (5.20 × 10^–28^m^3^ for C_28_), *k* is the Boltzmann constant (1.38 × 10^–23^m^2^ kg/s^2^ K), and λ is the molecular latent heat of crystallization.

Assuming spherical nuclei, the critical nucleus radius (r*) and the number of molecules in the critical nucleus (i*) are calculated using

13 $$r^{*}=\frac{2\gamma_{eff}v_{0}}{\lambda u}$$

14 $$i^{*}=\frac{{2bkT}_{e}}{{\lambda u}^{3}}$$

If assuming a cylindrical nucleus, the number of C_28_ molecules in a critically sized nucleus ( $i_{c_{28}}^{*}$ ) is determined by

15 $$i_{c_{28}}^{*}=\frac{r^{*}}{r_{c_{28}}}$$

where *r*_c28_ is the radius of C_28_ molecule based on a cylindrical geometry (2.16 × 10^–10^m^3^).

## Results and Discussion

### RA and IAA Aggregation

AFM imaging of RA and IAA samples at different concentrations in 6:4 DodecTol are shown in Figure 1. The IAA sample at 0.1 g/L shows the substantial formation of large, irregular, and interconnected particles (Figure 1a), while at the low concentration of 0.01 g/L, significantly fewer particles were seen with little interconnectivity (Figure 1b). The RA samples at 0.01 g/L also showed a dominance of very small particles (Figure 1d), however, less dependency on concentration was observed at 0.1 g/L RA in which the asphaltene particles appear discrete and slightly larger with generally spherical morphology (Figure 1c). In toluene, AFM imaging revealed flat, featureless surfaces with no visible asphaltene particles for RA. Only the IAA sample showed very few and relatively small particles at 0.1 g/L, with less seen at 0.01 g/L (Figure S6). The observed size differences between the IAA and RA samples are consistent with the findings of Qiao et al.^23^ and Ballard et al.^5^ who showed that both fractions aggregate more in more aliphatic solvents, but the IAA fraction has a tendency to aggregate/cluster in both aliphatic and aromatic solvents due to its higher oxygen and sulfur content, especially sulfoxide groups that promote strong polar interactions.

**Figure 1:**
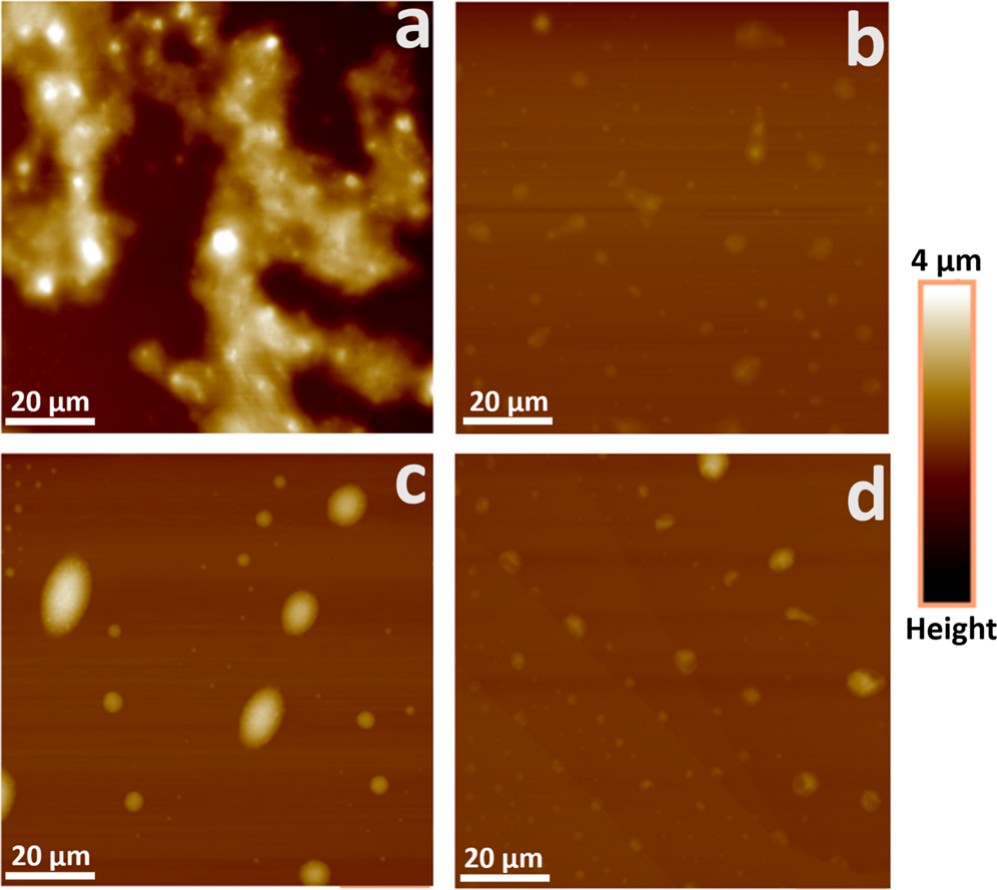
AFM images showing the particle sizes of RA or IAA subfractions taken from 6:4 DodecTol solution after 1 h aging time. (a) 0.1 g/L IAA, (b) 0.01 g/L IAA, (c) 0.1 g/L RA, and (d) 0.01 g/L RA. The liquid sample was placed on the AFM substrate (glass slide coated with freshly cleaved mica sheet) and then fast dried.

The observed particle sizes are important, because within the framework of the Yen–Mullins asphaltene size hierarchy,^4,23^ the presence of large asphaltene particles means that across the size hierarchy of molecules, nanoaggregates (dissolved) and particles (precipitated), the apparent equilibrium is shifted to the right, with molecules and nanoaggregates consumed to form the very large particles. However, for few small particles, or no observed particles, the apparent equilibrium shifts to the left, with more molecules and nanoaggregates remaining in solution. This balance, as will be discussed below, affects how the asphaltenes interact with C_28_ molecules, nuclei, and crystals.

### Effect of Solvent Type on C_28_ Solubility and Crystallizability

The equilibrium solubility temperature (*T*_e_) and supersolubility temperature (*T*_c,l_) determined from *T*_c_ and *T*_diss_ are shown in Figure 2a and summarized in Table 1 rows 1 to 4, along with the corresponding metastable zone widths (MSZWs) for C_28_ in 6:4 DodecTol and toluene. Additionally, the van’t Hoff plots and the resulting thermodynamic parameters for C_28_ in both solvents are presented in Figure 2b and Table 2 rows 1 and 4.

**Figure 2:**
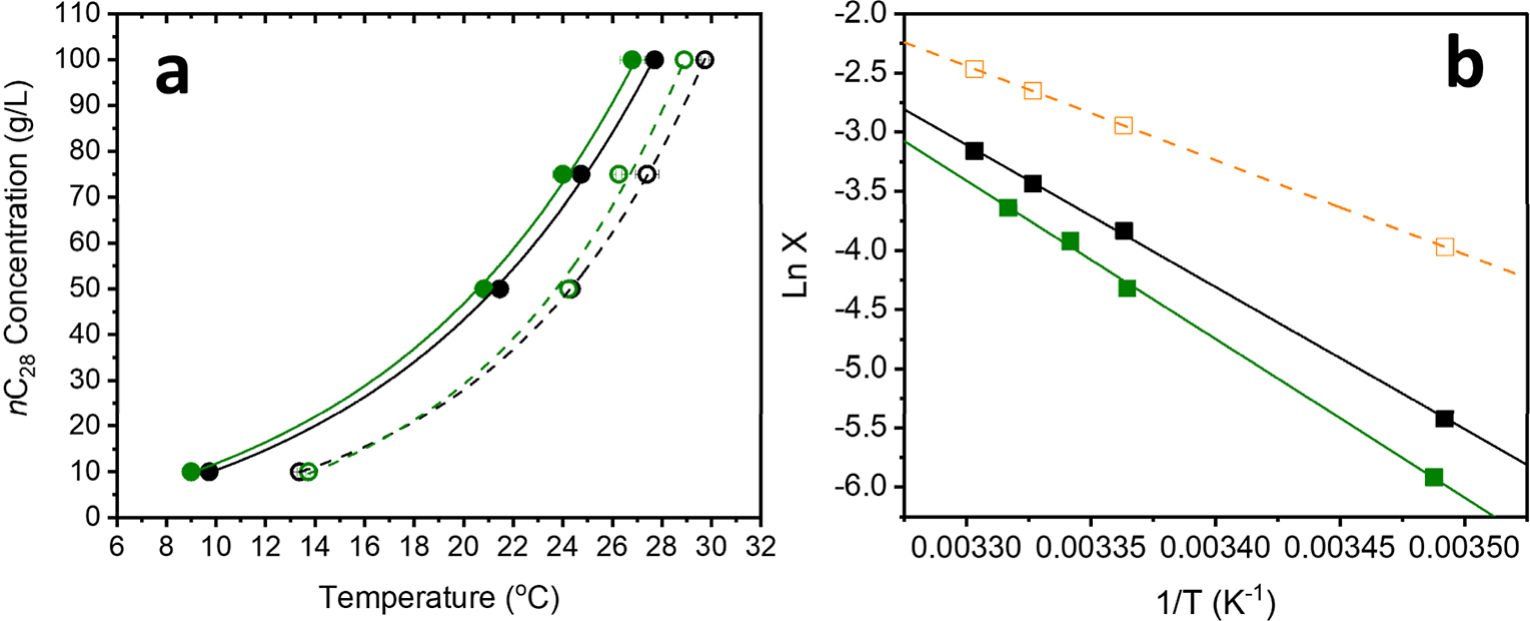
(a) Solubility (*T*_e_) curves of pure C_28_ in 6:4 DodecTol (○) and toluene (○(green)), and supersolubility (*T*_c,l_) curves of pure C_28_ in 6:4 DodecTol (●) and toluene (●(green)). (b) van’t Hoff plots of real *n*C_28_ solubility in 6:4 DodecTol (■) and toluene (■(green)). The dashed orange line represents the ideal octacosane solubility (□(orange)).

**Table 1:** Solubility (*T*_e_) and Supersolubility (*T*_c,l_) Data of Octacosane in 6:4 DodecTol or Toluene Solutions without and with RA or IAA at a Fixed C_28_:asphaltene Ratio (1000:1 wt/wt), Together with the Calculated MSZW Values Over Four Concentrations^a^

	C_28_ con. (g/L)	6:4 DodecTol					toluene				
		*T*_c,l_ (°C)	STD (°C)	*T*_e_ (°C)	STD (°C)	MSZW (°C)	*T*_c,l_ (°C)	STD (°C)	*T*_e_ (°C)	STD (°C)	MSZW (°C)
	pure C_28_										
1	10	9.45	0.24	13.37	0.10	3.92	9.00	0.07	13.72	0.08	4.72
2	50	21.45	0.11	24.34	0.07	2.89	20.81	0.20	24.22	0.26	3.41
3	75	24.73	0.18	27.60	0.47	2.87	24.28	0.10	26.26	0.11	1.98
4	100	27.71	0.17	29.73	0.16	2.02	26.80	0.10	28.51	0.11	2.51
	RA										
5	10	6.95	0.11	17.76	0.10	10.81	7.88	0.20	16.60	0.10	8.72
6	50	20.34	0.15	25.92	0.12	5.58	19.53	0.12	24.76	0.10	5.23
7	75	24.35	0.17	28.55	0.20	4.20	22.95	0.10	27.00	0.03	4.05
8	100	26.80	0.13	30.67	0.12	3.87	25.68	0.14	28.81	0.13	3.13
	IAA										
9	10	7.00	0.20	18.15	0.18	11.15	8.51	0.11	14.87	0.03	6.36
10	50	21.33	0.13	25.74	0.15	4.41	20.11	0.10	24.70	0.10	4.59
11	75	24.53	0.15	27.44	0.13	2.91	23.35	0.10	26.71	0.08	3.36
12	100	27.84	0.11	29.81	0.15	1.97	25.60	0.11	28.92	0.14	3.32

a The standard deviation is based on 12 scans. Note that the use of two decimal places in the table is intentional, as the standard deviations (i.e., measurement uncertainties) are very small, down to 0.03 °C in some cases. The Crystal-16 instrument has a temperature measurement accuracy of ±0.5 °C. Accordingly, temperature differences greater than 0.5 °C can be considered significant, whereas smaller differences fall within the instrument’s margin of error and should be interpreted as comparable.

**Table 2:** Thermodynamic Parameters and Activity Coefficients of C_28_, without and with RA or IAA Asphaltene Subfractions at 1000:1 wt/wt C_28_:asphaltene Ratio, Calculated from van’t Hoff Analysis of Solubility Data in 6:4 DodecTol or Toluene Solutions

	6:4 DodecTol					
	solute + Asp	Δ*H*_diss_ (kJ/mol K)	Δ*S*_diss_ (kJ/mol K)	Δ*H*_mix_ (kJ/mol K)	Δ*S*_mix_ (kJ/mol K)	activity coefficient (ϒ)
1	C_28_	101.55	0.30	33.30	0.10	2.10–4.60
2	C_28_ + RA	130.71	0.40	64.42	0.20	2.20–6.52
3	C_28_ + IAA	146.30	0.46	80.0	0.26	2.00–6.70
	toluene					
4	C_28_	104.80	0.34	45.50	0.14	1.95–4.75
5	C_28_ + RA	136.85	0.42	70.56	0.22	3.00–9.60
6	C_28_ + IAA	118.71	0.36	52.42	0.16	3.00–8.11

Figure 2a shows that the solubilities of C_28_ in 6:4 DodecTol and pure toluene are similar across the measured temperature range. This observation is consistent with prior findings by Jackson et al.^28^ and Provost et al.^40^ who demonstrated that for heavy *n*-alkanes (typically C_20_ and above), the nature of hydrocarbon solvents, whether linear, cyclic, or aromatic, has minimal effect on solubility. This behavior can be attributed to the inherently low polarity and weak self-association of *n*-alkanes, which leads to a dominant influence of solute–solute interactions over solvent–solute interactions. Since these long-chain alkanes exhibit similar van der Waals interaction profiles with nonpolar solvents, their solubility remains relatively insensitive to subtle differences in solvent structure within the hydrocarbon class.

The MSZW of C_28_ in toluene was slightly larger than in the 6:4 DodecTol mixture, indicating a reduction in crystallizability. This difference is attributed to a modest shift in the supersolubility temperature (*T*_c,l_) toward lower values across all concentrations studied (Figure 2a and Table 1, rows 1–4). One possible explanation is that toluene forms a more disordered and potentially more polar solvated layer around C_28_ molecules, due to its higher dipole moment compared to 6:4 DodecTol. This disordered solvation environment may hinder the diffusive transport of C_28_ molecules into prenucleation clusters and reduce their attachment frequency to growing nuclei.^27,28^ Notably, the average MSZW observed in both solvents was relatively narrow (∼3 °C), consistent with previous studies on alkane crystallization.^27,41^

### Effect of Solvent Type on C_28_ Solution Thermodynamics

The van’t Hoff analysis provides valuable insight into the thermodynamic behavior of C_28_ alkane solutions in different solvents. The van’t Hoff plots (Figure 2b) show a strong linear relationship between modeled and experimental solubility data, indicating that the solid phase of C_28_ remains unchanged in both 6:4 DodecTol and toluene. In both solvents, the solubility deviates from ideal behavior as shown by the activity coefficients γ being greater than 1 with similar values (Table 2 rows 1 and 4).

The rise in the enthalpy of dissolution and activity coefficients (Table 2) further reveal a preference for solute–solute interactions over solute–solvent interactions, meaning that the process is endothermic. C_28_ molecules tend to associate with each other due to strong lateral vdW forces along their long chains. Solvation by either toluene or 6:4 DodecTol disrupts these interactions, but the smaller size of the solvent molecules results in weaker vdW forces between solute and solvent compared to solute–solute interactions.^28^ This behavior is reflected in the positive enthalpy of dissolution (Δ*H*_diss_) values reported in Table 2 rows 1 and 4.

### Effect of RA or IAA on C_28_ Solubility and Crystallizability

The equilibrium solubility (*T*_e_) and supersolubility (*T*_c,l_) temperatures as well as the supersaturation ratios (*S*) of C_28_ without and with RA or IAA in 6:4 DodecTol or toluene are shown in van’t Hoff coordinates in Figures 3 and 4 and the equilibrium data are provided in Table 1, along with their corresponding MSZW.

**Figure 3:**
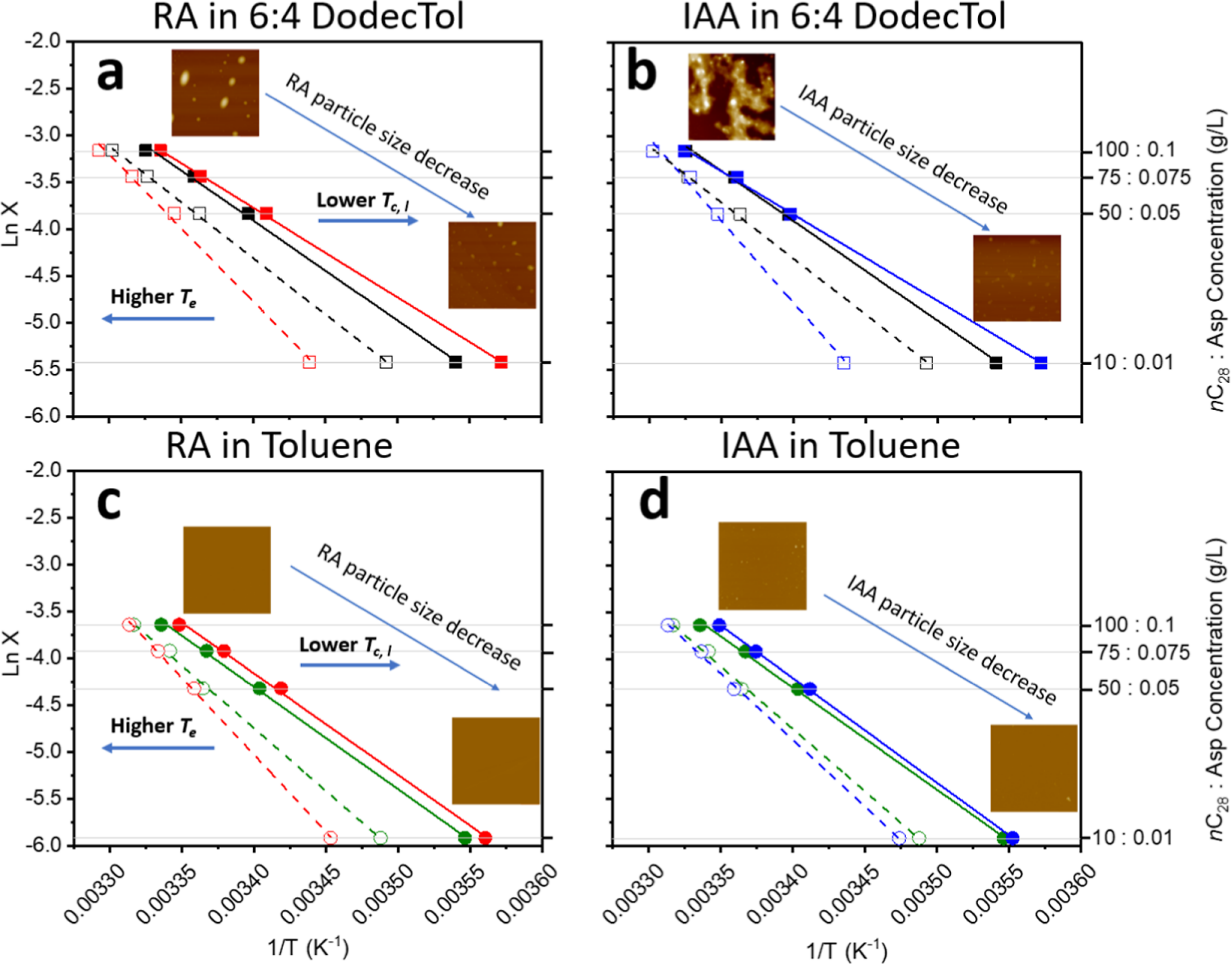
Equilibrium solubility (*T*_e_) and supersolubility (*T*_c,l_) temperatures shown plotted in van’t Hoff coordinates as (1/*T*) for C_28_ solutions without and with asphaltenes. (a,b) compare 6:4 DodecTol solutions without and with RA or IAA, (c,d) compare toluene solutions without and with RA or IAA. The C_28_:asphaltene ratio was fixed at 1000:1 wt/wt. (□) and (■) are the solubility and supersolubility curves of C_28_ in 6:4 DodecTol, respectively. (□(red)) and (■(red)) are the solubility and supersolubility curves of C_28_ with RA in 6:4 DodecTol, respectively. (□(blue)) and (■(blue)) are the solubility and supersolubility curves of C_28_ with IAA in 6:4 DodecTol, respectively. (○(green)) and (●(green)) are the solubility and supersolubility curves of C_28_ in toluene, respectively. (○(red)) and (●(red)) are the solubility and supersolubility curves of C_28_ with RA in toluene, respectively. (○(blue)) and (●(blue)) are the solubility and supersolubility curves of C_28_ with IAA in toluene, respectively. The inset AFM images compare RA or IAA particle size variations when decreasing asphaltene concentration from 0.1 g/L to 0.01 g/L. The data was plotted in van’t Hoff coordinates to better observe the differences between the pure and impure samples.

**Figure 4:**
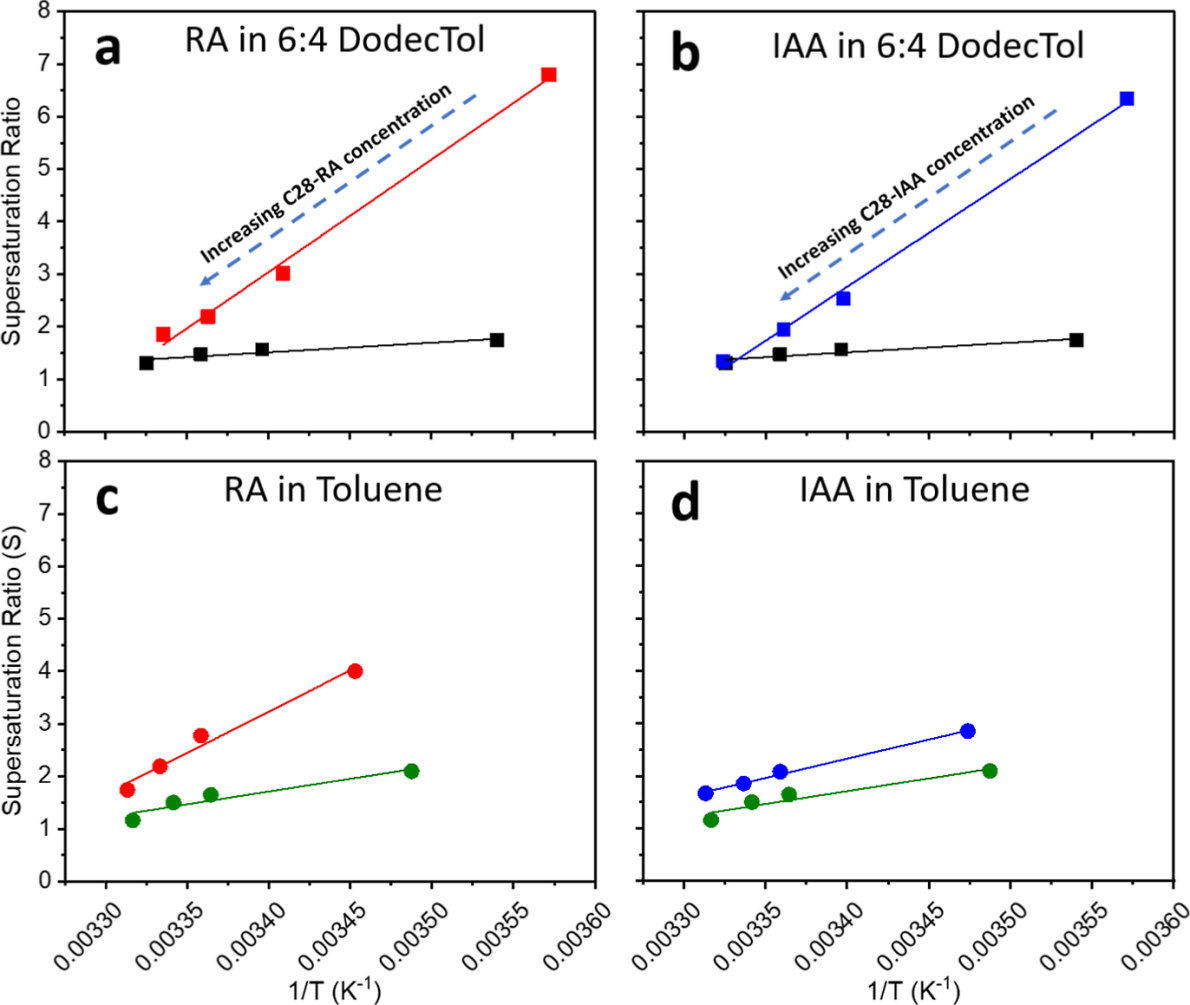
Equilibrium supersaturation ratios (*S*) in van’t Hoff coordinates as (1/*T*) for C_28_ in 6:4 DodecTol or toluene solutions without and with asphaltenes. (a,c) compare supersaturation ratio behavior of systems with and without RA in 6:4 DodecTol and toluene, respectively. (b,d) compare supersaturation ratio behavior of systems with and without IAA in 6:4 DodecTol and toluene, respectively. Note that the C_28_:asphaltene ratio is fixed at 1000:1 wt/wt. (■) is the supersaturation of C_28_ in 6:4 DodecTol, (■(red)) is the supersaturation of C_28_ with RA in 6:4 DodecTol, (■(blue)) is the supersaturation of C_28_ with IAA in 6:4 DodecTol, (●(green)) is the supersaturation of C_28_ in toluene, (●(red)) is the supersaturation of C_28_ with RA in toluene, and (●(blue)) is the supersaturation of C_28_ with IAA in toluene.

When RA or IAA subfractions were added to a 6:4 DodecTol mixture, the solubility temperature (*T*_e_) increased significantly at the lowest concentration of 10 g/L C_28_ + 0.01 g/L RA or IAA, as shown in Table 1 and indicated by the lower 1/T values in Figures 3a and b. However, this effect diminished as the concentration of solute-asphaltene increased in which the solubility temperature (*T*_e_) only increased by ∼1 °C at the highest concentration of 100 g/L C_28_ + 0.1 g/L RA (Table 1, rows 4 and 8), while becoming negligible at 100 g/L C_28_ + 0.1 g/L IAA (Table 1, rows 4 and 12). This behavior suggests that RA and IAA become less effective at modifying solubility as the C_28_-asphaltene concentration increases with IAA being almost inert at the highest concentration of 100 g/L C_28_ + 0.1 g/L IAA. The concentration-dependent behavior can be rationalized against the apparent equilibrium of the asphaltenes size hierarchy.

As the asphaltenes concentration increases (100 g/L C_28_ with 0.1 g/L RA or IAA), larger asphaltene particles are formed (inset images in Figure 3a,b), thereby reducing the number of molecules and nanoaggregates in solution available to interact with the C_28_ molecules. However, at the lower concentration of 10 g/L C_28_ with 0.01 g/L RA or IAA, the apparent equilibrium shifts, and there will be an excess of asphaltene molecules and nanoaggregates in solution to interact with C_28_.

Similarly, the supersolubility temperature (*T*_c,l_) of 6:4 DodecTol solutions was observed to decrease as the concentration of RA or IAA decreased (Table 1 and Figures 3a and b). This trend is influenced by the increased number of asphaltene molecules/nanoaggregates at the lower concentration, enhancing the ability to inhibit crystallization. However, at the higher concentration, the effect is diminished, as understood from the apparent equilibrium shift of the size hierarchy, with these findings being consistent with our previous study,^12^ which showed that increasing the concentration of RA or IAA up to 1 g/L had a negligible effect on the crystallization onset (WAT) of 100 g/L C_28_ in 6:4 DodecTol.

The observed behavior of both *T*_e_ and *T*_c,l_ led to a broadening of the MSZW of the solute, indicating reduced crystallizability of C_28_ when RA or IAA subfractions were added to 6:4 DodecTol (Table 1). This effect was most pronounced at the lowest concentration studied. Again, the crystallizability of 100 g/L C_28_ when adding 0.1 g/L IAA remained almost unchanged (Table 1) due to the dominance of the large asphaltene particles which consume asphaltene molecules and nanoaggregates, and so become less effective at interacting with solute molecules. The broadening of the MSZW suggests an increase in the free energy barrier required for nucleation, meaning that nucleation could only occur once a sufficiently high level of supersaturation was achieved. Figure 4a,b clearly show a linear increase in C_28_ supersaturation with decreasing C_28_-asphaltene concentrations in the presence of RA or IAA. This crystallization inhibition likely reflects a reduction in the nucleation and growth kinetics of C_28_, possibly due to chemical and steric interactions between the solute and additive molecules within the crystallizing solution.^18,19,21,28^ The kinetics of nucleation will be discussed in the following section.

In toluene, the thermodynamic temperatures (*T*_e_ and *T*_c,l_) followed similar trends to those observed in 6:4 DodecTol, but the effects were significantly less pronounced (Figures 1d and 4c).

This is because asphaltenes in toluene are better solubilized (Figure S6), hence there is less of an effect on shifting the size hierarchy, with molecules and nanoaggregates remaining abundant. This observation is supported by Ballard et al.,^42^ who conducted molecular simulation studies showing that the abundance-weighted mean interaction energies of RA or IAA with toluene were at least 20% higher than with heptane (a solvent similar to dodecane).

The solubility results suggest that solution thermodynamics are influenced by the asphaltenes, which disrupt the natural solute–solvent and solute–solute interactions. As shown in the van’t Hoff analysis (Figure 5), the solution behavior becomes increasingly nonideal in the presence of asphaltenes, leading to higher activity coefficients and an increase in the total enthalpy of dissolution (Δ*H*_diss_) (Table 2). These findings are consistent with the idea that asphaltene molecules and nanoaggregates strongly bind to the solute through noncrystallographic vdW interactions,^21^ which suggests that greater solvation energy is required to overcome these C_28_-asphaltene interactions. This interpretation aligns with the findings of Xu et al.,^43^ who confirmed that solute–additive interactions can reduce solubility, as reflected by an increase in *T*_e_.

**Figure 5:**
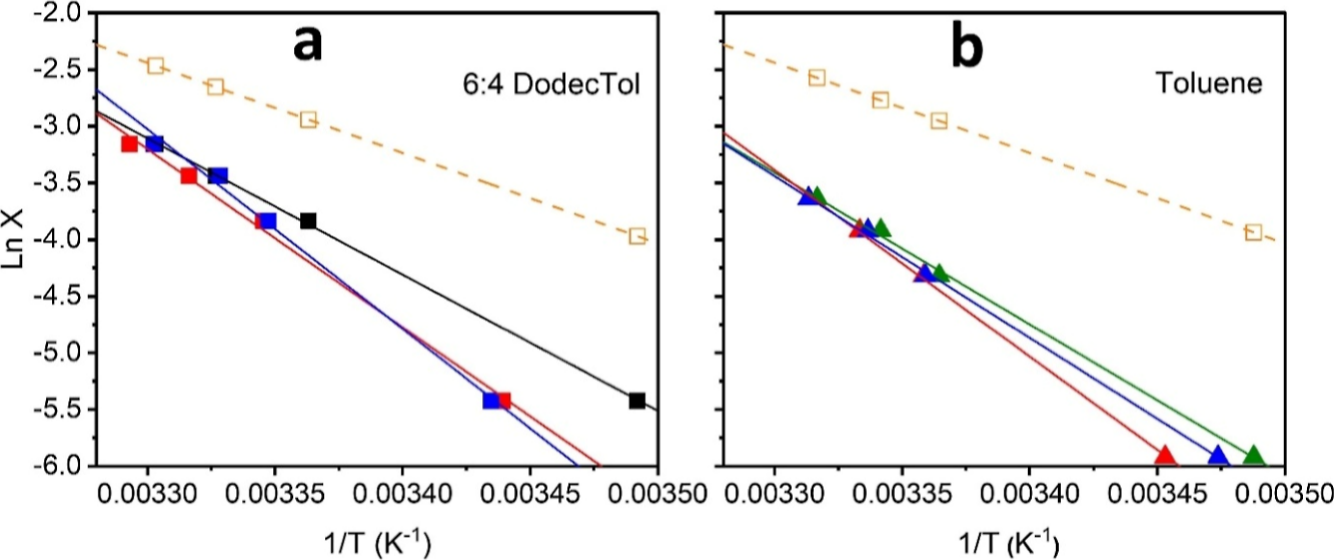
van’t Hoff plots of real C_28_ solubility without and with asphaltenes in (a) 6:4 DodecTol, and (b) toluene. (■) and (▲(green)) are real C_28_ solubility in 6:4 DodecTol or toluene, respectively. (■(red)) and (▲(red)) are real C_28_ solubility with RA in 6:4 DodecTol or toluene, respectively. (■(blue)) and (▲(blue)) are real C_28_ solubility with IAA in 6:4 DodecTol or toluene, respectively. (□(orange)) represents the ideal C_28_ solubility.

### Effect of RA or IAA on C_28_ Nucleation Kinetics

Figure 6 shows that the calculated nucleation mechanism parameter (ω) is >3 for all solutions, indicating that crystallization predominantly follows a progressive nucleation (PN) pathway. As the supersaturation driving force increases with the addition of RA or IAA (Figure 5), the nucleation becomes even more progressive, with the most pronounced effect observed at the lowest solute-to-asphaltene concentrations. In contrast, when asphaltene subfractions are dissolved in toluene, they exhibit a weaker tendency to promote progressive nucleation (Figure 6b). This behavior likely reflects a preference for asphaltene–solvent interactions over asphaltene–solute interactions, which is consistent with the solubility and crystallizability data. The corresponding ln *q* vs ln *u*_c_ or *u*_c_ plots used to derive the nucleation kinetics are provided in Figures S9–S11.

**Figure 6:**
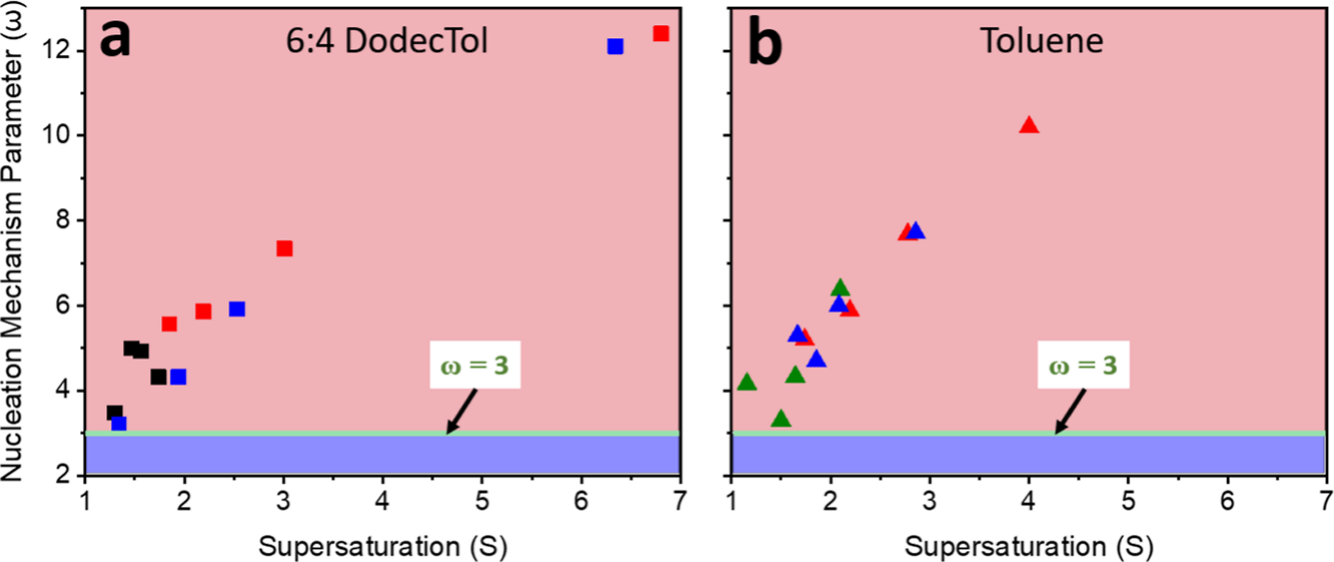
Change in nucleation mechanism parameter (ω) with supersaturation when adding RA or IAA subfractions at C_28_:asphaltene ratio of 1000:1 wt/wt in (a) 6:4 DodecTol and (b) toluene. (■) and (▲(green)) are the nucleation parameters for pure C_28_ in 6:4 DodecTol or toluene, respectively. (■(red)) and (▲(red)) are the nucleation parameters for C_28_ with RA in 6:4 DodecTol or toluene, respectively. (■(blue)) and (▲(blue)) are the nucleation parameters for C_28_ with IAA in 6:4 DodecTol or toluene, respectively.

The effective interfacial tension (γ_eff_) increased significantly with the addition of RA or IAA at low solute-to-asphaltene concentrations, with a diminished effect observed at higher concentrations, becoming negligible for IAA at the highest concentration of 100 g/L C_28_ + 0.1 g/L IAA (Figure 7). This trend closely parallels the behaviors seen in the solubility and crystallizability data, with the shift toward more progressive nucleation suggesting that nucleation in these systems is more thermodynamically controlled. This trend also corresponds to a slight increase in the critical nucleus size (r*), the ratio of r* to the radius of a C_28_ molecule (r*/r_c28_), and the number of molecules in the critical nucleus (i*) in the presence of asphaltenes (Table S3 in the Supporting Information). A similar, though less pronounced, trend was also observed in toluene, see Table S4 in the Supporting Information.

**Figure 7:**
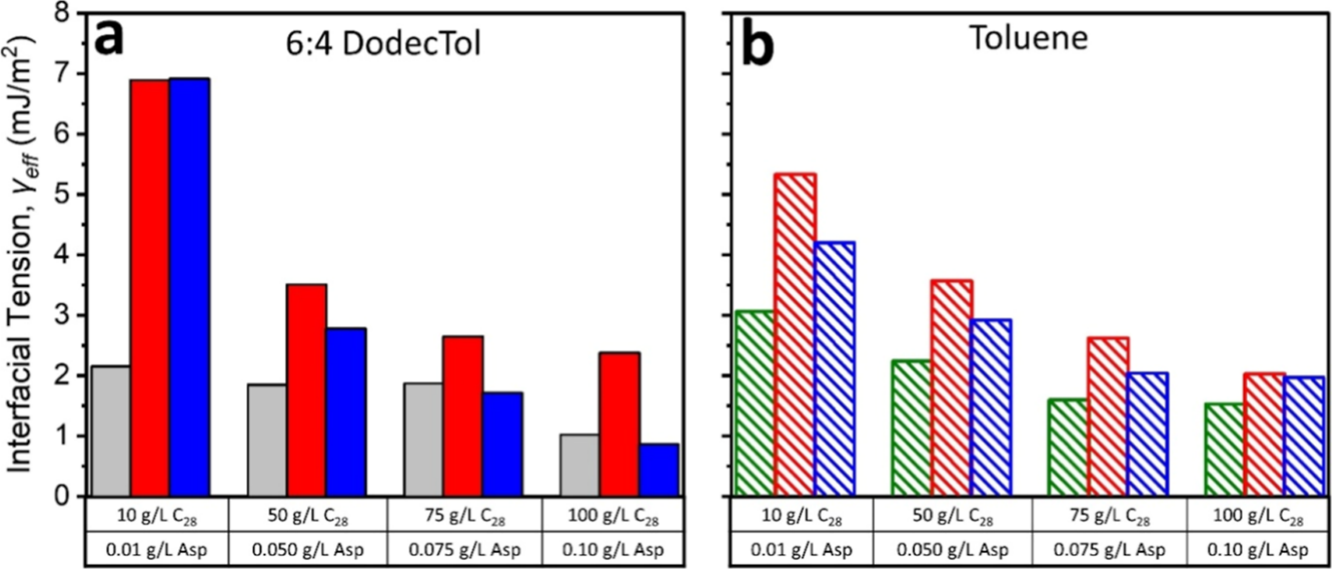
Change in effective interfacial tension (γ_eff_) when adding RA or IAA subfractions at C_28_:asphaltene ratio of 1000:1 wt/wt in (a) 6:4 DodecTol and (b) toluene. 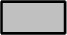 and 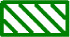 are γ_eff_ values of pure C_28_ in 6:4 DodecTol or toluene, respectively. 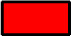 and 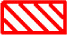 are γ_eff_ values of C_28_ with RA in 6:4 DodecTol or toluene, respectively. 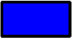 and 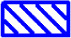 are γ_eff_ values of C_28_ with IAA in 6:4 DodecTol or toluene, respectively.

This behavior is consistent with the adsorption of asphaltene molecules and nanoaggregates onto growing C_28_ prenucleation clusters, where vdW interactions between alkyl chains promote attachment and introduce steric hindrance. These effects make it more difficult for diffusing solute molecules to attach to the surface, thereby hindering the formation of a critically sized C_28_ nucleus. As a result, the critical nucleus size (r*) increases, meaning that a greater number of solute molecules is required to initiate nucleation.^18,43^

### Crystallization and Potential Inhibition Mechanisms

It is widely accepted that crystallization from solution follows a two-step process: nucleation, the initial formation of a crystal, followed by crystal growth. These two processes can occur simultaneously and are both influenced by the degree of supersaturation.^44^ In the case of alkanes, nucleation and crystal growth are relatively fast, making it difficult to determine which step is rate-limiting. Therefore, the observed increases in the MSZW (Figure 3) and supersaturation (Figure 4) in the presence of RA or IAA are likely due to asphaltene-mediated disruption of one or both of these processes.^45^ Building on this two-step model, a mechanistic framework can be proposed to better understand how asphaltene subfractions may alter the crystallizability of C_28_.

#### Step 1: Nucleation Inhibition

When the concentration of the asphaltene molecules and nanoaggregates exceeds that of the solute, as in the case of 10 g/L C_28_ with 0.01 g/L RA or IAA, these molecules and nanoaggregates interact with solute molecules via vdW forces in the liquid phase. They disrupt the natural solute–solute and solute–solvent interactions, as reflected by the increased enthalpy of dissolution (Table 2).^36^ During nucleation, the presence of C_28_-asphaltene motifs likely suppresses the formation of three-dimensional C_28_ clusters due to steric hindrance between the asphaltene and alkane molecular structures. In such systems, these asphaltene motifs are likely to attach to the surface of prenucleation C_28_ clusters, resulting in the formation of larger, less stable critical nuclei. This behavior is consistent with the observed shift toward a more progressive nucleation mechanism (Figure 6), as well as the increases in interfacial tension and the number of molecules in the critical nucleus (Figure 7, Tables S3 and S4).

Additionally, steric effects hinder the mobility of diffusing C_28_ molecules and reduce their ability to attach to growing clusters.^21^ As a result, nucleation can only proceed once a sufficiently high supersaturation is achieved to overcome the elevated energy barrier introduced by C_28_-asphaltene interactions. The effect of steric hindrance is supported by the increased degree of supersaturation shown in Figure 4. The proposed nucleation inhibition mechanism is illustrated in the lower schematic of Figure 8.

**Figure 8:**
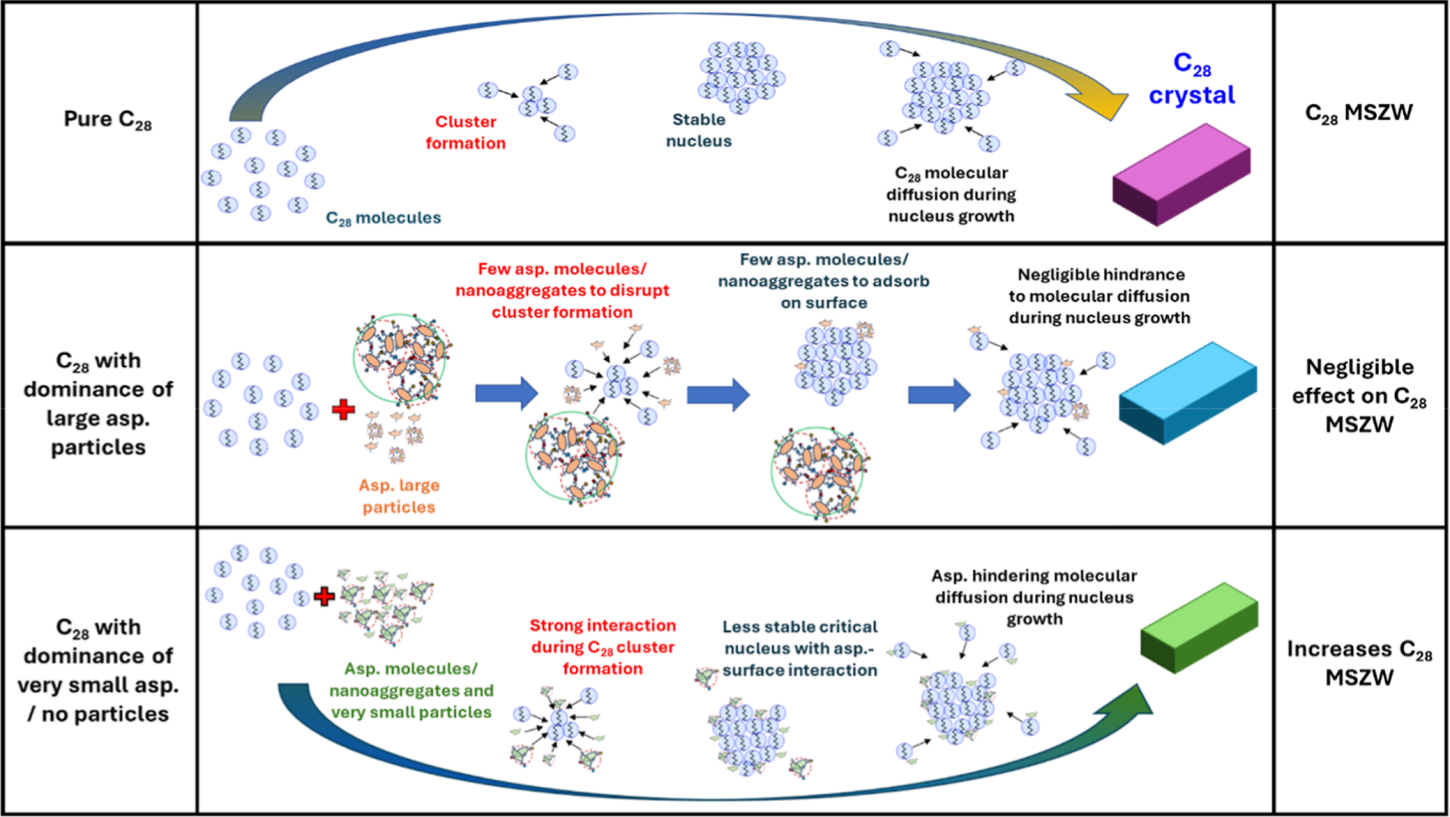
Schematic comparing the mechanisms by which asphaltenes influence the nucleation step of C_28_, in the presence of predominantly very small asphaltene particles (bottom) or large asphaltene particles (middle), with that of the pure C_28_ system (top).

Conversely, when the concentration of the asphaltene molecules and nanoaggregates is much lower than that of the solute, as in 100 g/L C_28_ with 0.1 g/L RA or IAA in 6:4 DodecTol, the likelihood of significant asphaltene–alkane interactions diminishes. This behavior is due to the predominance of larger asphaltene particles, which are less effective at interfering with the three-dimensional assembly of the alkane solute. As a result, the influence on crystallization becomes negligible, consistent with the minimal change observed in the MSZW for this system. This scenario is depicted in the middle schematic of Figure 8.

#### Step 2: Crystal Growth Inhibition

In this model, once nuclei are formed, they grow into crystals through faceting along habit planes via two-dimensional molecular self-assembly. The (*h k 0*) planes are inherently unstable at high supersaturation levels,^46^ becoming rough at the molecular scale. This roughness allows asphaltenes to become trapped on the crystal surface.^46,47^ Under such conditions, such as in the 10 g/L C_28_ + 0.01 g/L RA or IAA solution, asphaltene molecules and nanoaggregates are expected to adsorb onto and cover a large surface area of the (*h k 0*) faces, forming a porous asphaltene layer. This adsorbed layer likely blocks active growth sites, hindering the attachment of diffusing C_28_ molecules to the growing crystal faces. As a result, more energy is required for C_28_ crystallization, leading to an increase in the MSZW. This adsorption mechanism was observed in our previous study,^12^ where RA and IAA subfractions were shown to reduce the size of C_28_ needle-like crystals, confirming their crystal growth inhibition effect. This mechanism is illustrated in Figure 9.

**Figure 9:**
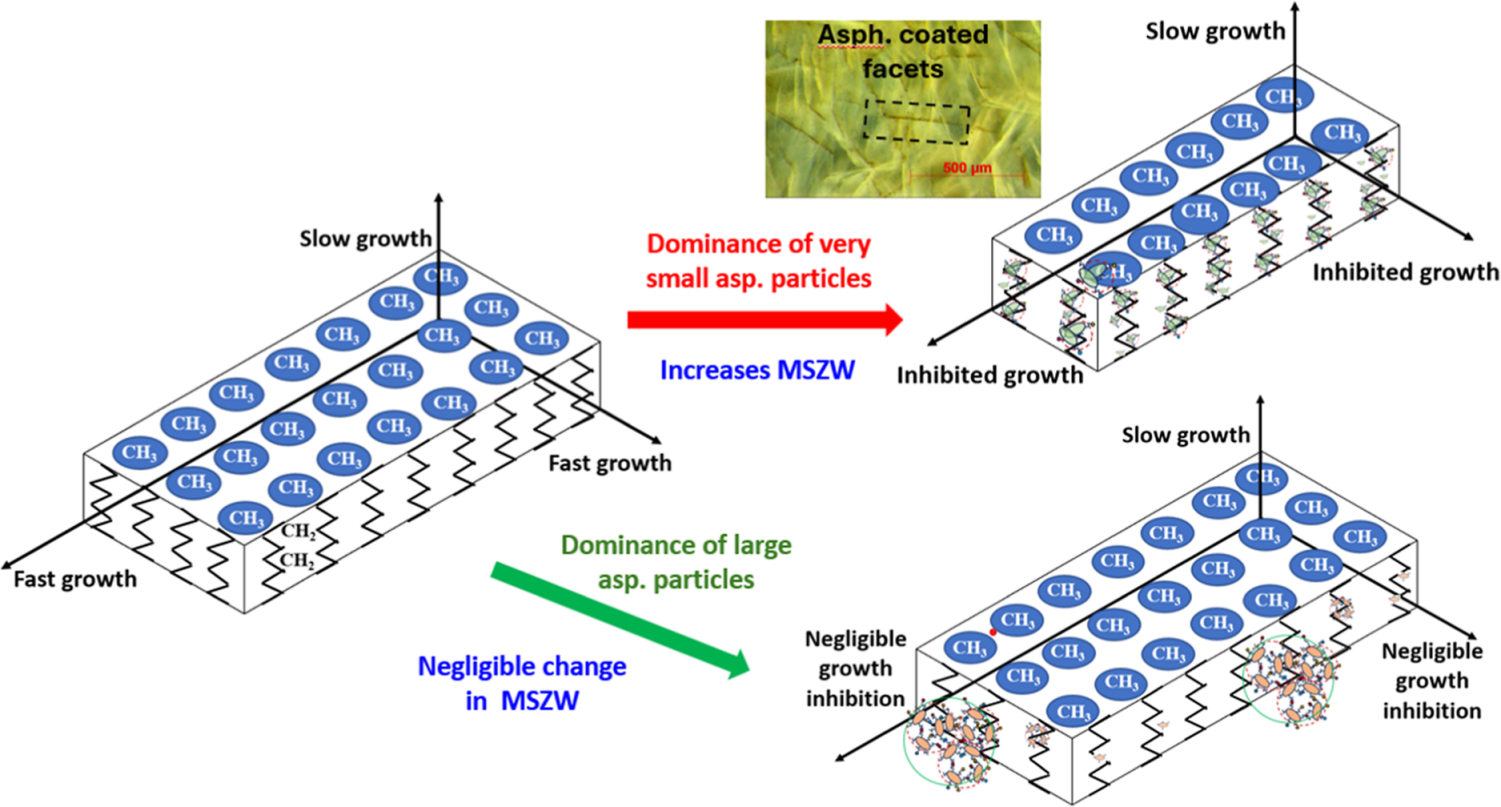
Schematic illustrating the growth inhibition mechanism under conditions dominated by very small asphaltene particles and nanoaggregates (e.g., 10 g/L C28 + 0.01 g/L RA) or by large asphaltene particles (e.g., 100 g/L C_28_ + 0.1 g/L IAA). The schematic was adapted from Roberts.^48^ The inset image shows a dark field microscopy of C_28_ gel network highlighting layers of asphaltene motifs coating C_28_ crystals.

In contrast, in solutions where the concentration of asphaltene molecules and nanoaggregates is significantly lower than that of the solute, due to the predominance of large asphaltene particles, their ability to inhibit crystal growth is expected to be negligible. These larger particles are entropically restricted and less likely to bind effectively to the (*h k 0*) faces, resulting in minimal surface coverage. Consequently, their impact on C_28_ crystallizability is limited, as reflected by the negligible changes observed in Table 1. This scenario is also illustrated in Figure 9.

## Conclusions

A model waxy-asphaltenic oil system, based on a C_28_ solute and exhibiting varying degrees of aromaticity, was investigated to better understand the influence of asphaltene subfractions, with differing polarities and interfacial activities, on the nucleation stage of wax crystallization. This was achieved by characterizing solubility, solution thermodynamics, metastable zone width (MSZW), and nucleation kinetics parameters. While previous studies have primarily focused on the effect of asphaltenes on the wax appearance temperature (WAT), few have explored their impact on the specific crystallization steps of nucleation and growth. In this study, asphaltenes were extracted from a heavy crude oil and further fractionated into two subfractions: weakly interfacially active (RA) and strongly interfacially active (IAA), each exhibiting distinct physicochemical properties. The IAA fraction is more polar, largely due to their higher content of heteroatoms such as sulfur and oxygen, and tend to form larger particles in solution compared to the RA fraction.

In the presence of either RA or IAA at low concentrations, specifically, at a fixed C_28_-to-asphaltene ratio of 1000:1 (w/w), the solubility temperature (*T*_e_), MSZW, and supersaturation ratio (*S*) increased significantly. This effect was inversely proportional to the overall C_28_-asphaltene concentration at the constant ratio. For example, in 6:4 DodecTol, the MSZW increased by ∼7 °C at a concentration of 10 g/L C_28_ with 0.01 g/L RA or IAA. However, the increase was ∼1 °C at 100 g/L C_28_ with 0.1 g/L RA, and no significant change was observed for the same concentration with IAA. This behavior is consistent with a transition in asphaltene particle size, from predominantly very small particles (with more asphaltene molecules and nanoaggregates remaining in solution) at low concentrations to larger particles at higher concentrations (with fewer molecules and nanoaggregates in solution). The larger IAA particles interact less effectively with C_28_ because of size mismatches, making them inherently less likely to bind to C_28_ molecules, and this effect is compounded by the reduced number of asphaltene molecules and nanoaggregates in solution. As a result, the IAA fraction showed a reduced interaction with C_28_ at the highest concentration tested (100 g/L C_28_ + 0.1 g/L IAA) compared to RA.

A 2-step crystallization model was proposed to describe the mechanisms by which asphaltene subfractions inhibit crystallization; a summary of this model is presented in Table 3. Additionally, both RA and IAA subfractions altered the solution thermodynamics, making the system less ideal than in the absence of asphaltenes. This decrease in the solution’s ideality led to an increase in the total enthalpy of dissolution (Δ*H*_diss_).

**Table 3:** Proposed 2-Step Crystallization Inhibition Model

crystallization step	mechanisms
step 1 – birth of a crystal (3D nucleation)	1. Suppressing 3D cluster formation and forming unstable nuclei with blocked nucleation sites.2. Inhibiting C_28_ molecules transfer from bulk fluid to nuclei surface.
step 2 – crystal growth (2D nucleation)	1. Binding of asphaltenes onto C_28_ crystal growth surfaces and consequently blocking the growth sites.

Future research should investigate the crystal growth mechanisms of C_28_ in the presence of RA or IAA subfractions, as this represents another important factor to consider when evaluating the influence of each subfraction on wax properties within the broader context of flow assurance. This could be achieved by measuring the supersaturation-dependent growth rates of individual crystal faces and analyzing the resulting crystal morphology. The study could also be extended to include alkanes of varying chain lengths and polarities, as well as mixtures thereof.

## Supplementary Material



## Notes

Chemical characteristics of asphaltenes, sample preparation methodology, crystal 16 temperature calibration curves, thermodynamic properties of C28 melt, photographic images of asphaltene aggregation in sample vials, AFM aggregate size analysis of RA and IAA in toluene, C_28_ polythermal plots, and the KBHR data of the nucleation mechanism and kinetics (PDF)
