# Supplementary material for: Influence of Strongly and Weakly Interfacially Active Asphaltene Particles on Solubility and Crystallizability of Octacosane (C28) Model Oils
Source: Cryst Growth Des. 2025 Oct 7;25(21):8990–9002. doi: 10.1021/acs.cgd.5c00801 (PMC12593340; doi:10.1021/acs.cgd.5c00801)
Supplement: Supplementary file 1 [file cg5c00801_si_001.pdf]

Supplementary Information

Influence of strongly and weakly interfacially active asphaltene particles on solubility and crystallisability of octacosane (C_{28}) model oils

Abdulraouf Ali[†], Ghinwa Yaghy[†], Alexander Jackson[†], Chris S. Hodges[†], Thibaut V.J. Charpentier[††], Kevin J. Roberts[†] and David Harbottle*[†]

[†]School of Chemical and Process Engineering, University of Leeds, Leeds, LS2 9JT, UK
[††]Baker Hughes, Oilfield Services, Liverpool, L33 7TQ, UK

S1. Asphaltenes characterization

The elemental composition of the three asphaltene fractions (WA, RA, and IAA) was determined using EA112 Flash analyzer (CHNS/O). As per the manufacturer's recommended standard practice, 2 mg of each fraction was analyzed, with the mass range found to have no effect on the sample composition. The results reported were the average of multiple independent runs to minimize sampling and analysis error.

Table S 1. Elemental composition of WA, RA, and IAA.

Asp. type	C (wt %)	H (wt %)	N (wt %)	S (wt %)	O (wt %)	H/C	Total (wt %)
WA	83.65±0.6	7.26±0.03	1.28±0.2	3.41±0.78	2.42±0.61	1.04	98.02
RA	83.33±-1.38	7.28±0.26	1.37±0.08	3.40±0.75	2.56±0.39	1.04	97.94
IAA	74.41±0.32	7.08±0.66	1.51±-0.17	4.03±0.59	3.39±0.12	1.14	90.42

To characterize the polar groups contained in the asphaltene subtractions, FTIR spectra were obtained using a Nicolet iS10 FTIR spectrometer (ThermoScientific) equipped with an attenuated total reflection (ATR) sampling accessory in the spectral range between 4000 and 500 cm^{-1} at a spectral resolution of 2 cm^{-1}. A single spectrum was collected from a total of 32 scans. All FTIR spectra were normalized with respect to the strongest adsorption peak of the aliphatic C−H stretching vibrations between 2780 and 3000 cm^{-1}.

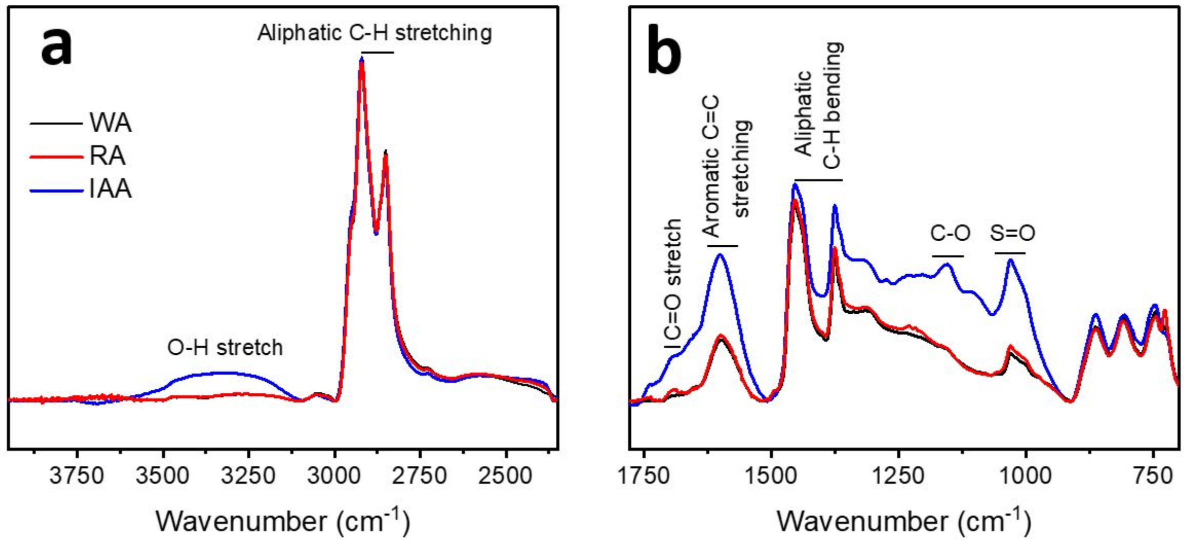

Figure S1. Normalized FTIR spectra of WA, RA and IAA (a) high wavenumber (2300 – 4000 cm^{-1}) (b) low wavenumber (700 – 1770 cm^{-1}).

S2. Sample preparation procedure

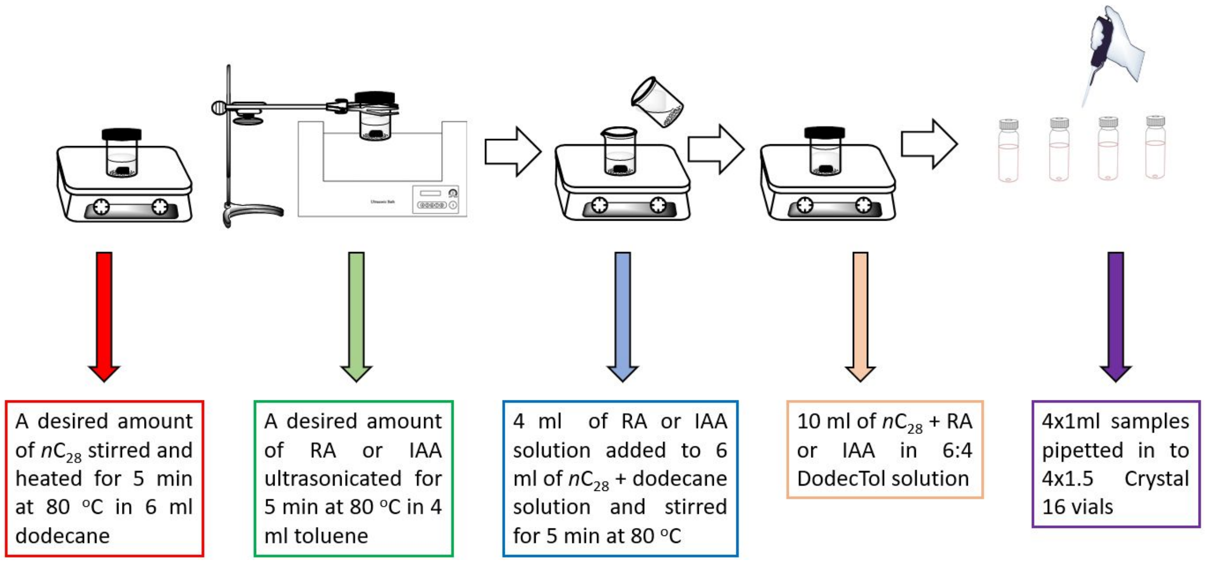

Figure S2. Schematic workflow showing the preparation procedure of 10 mL of C_{28} + RA or IAA in 6:4 DodecTol solution.

S3. Crystal 16 temperature calibration curves

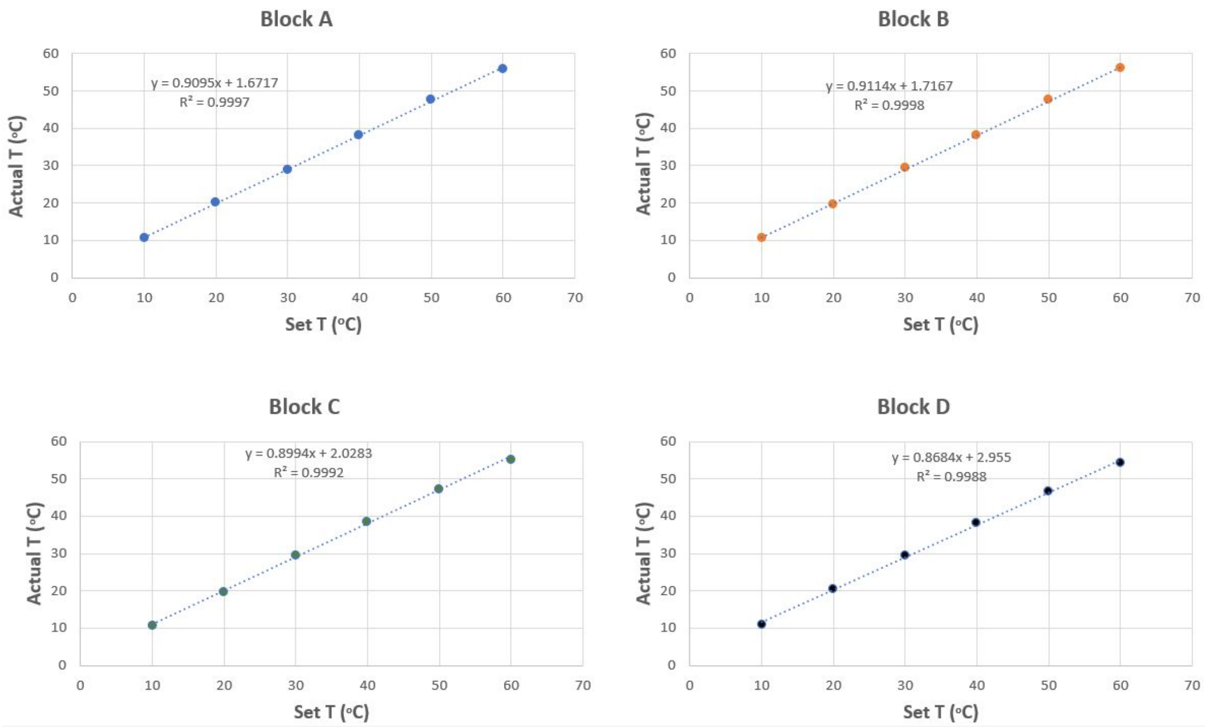

Figure S3. Temperature calibration curves of the 4 blocks used in the Crystal 16 unit in 6:4 DodecTol. Note that this is also valid for toluene.

S4. Melting Points and Enthalpies of Fusion

Table S2. Enthalpy of fusion and melting points of *n*-octacosane (nC_{28})

**Alkane Type**	**ΔH_{fus} (J/mol)**	**Tm (°C)**
**C_{28}**	66290 ± 32	58 ± 0.10

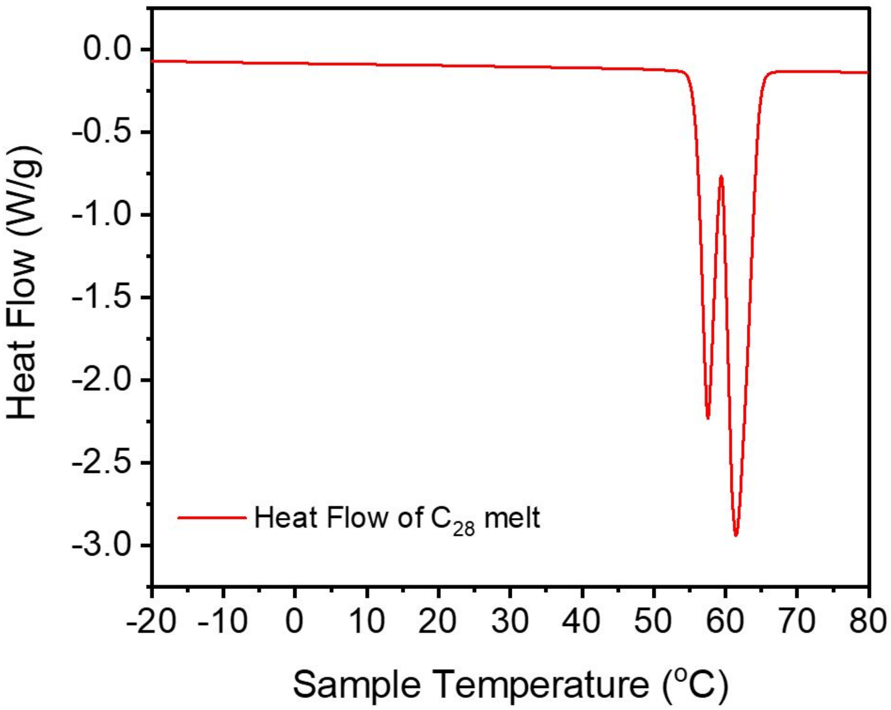

Figure S4. DSC curves of the melting of C_{28} obtained by heating at 5 °C/min.

It can be noted that the DSC scan of C_{28} shows two peaks. This is attributed to the fact that C_{28} can exist in monoclinic (M011, space group P21/a) orthorhombic (O_p, space group Pca21) and R_{IV} rotator phases in different temperature ranges. It is because if two phases (M and O_p) occur, most probably two solid-solid (s-s) phase transition peaks may be observed in the DSC curves[1]. According to Wang et al. [1], the low temperature phase should be the monoclinic phase and the melting point was taken at that peak.

S5. Aggregation behavior of RA and IAA

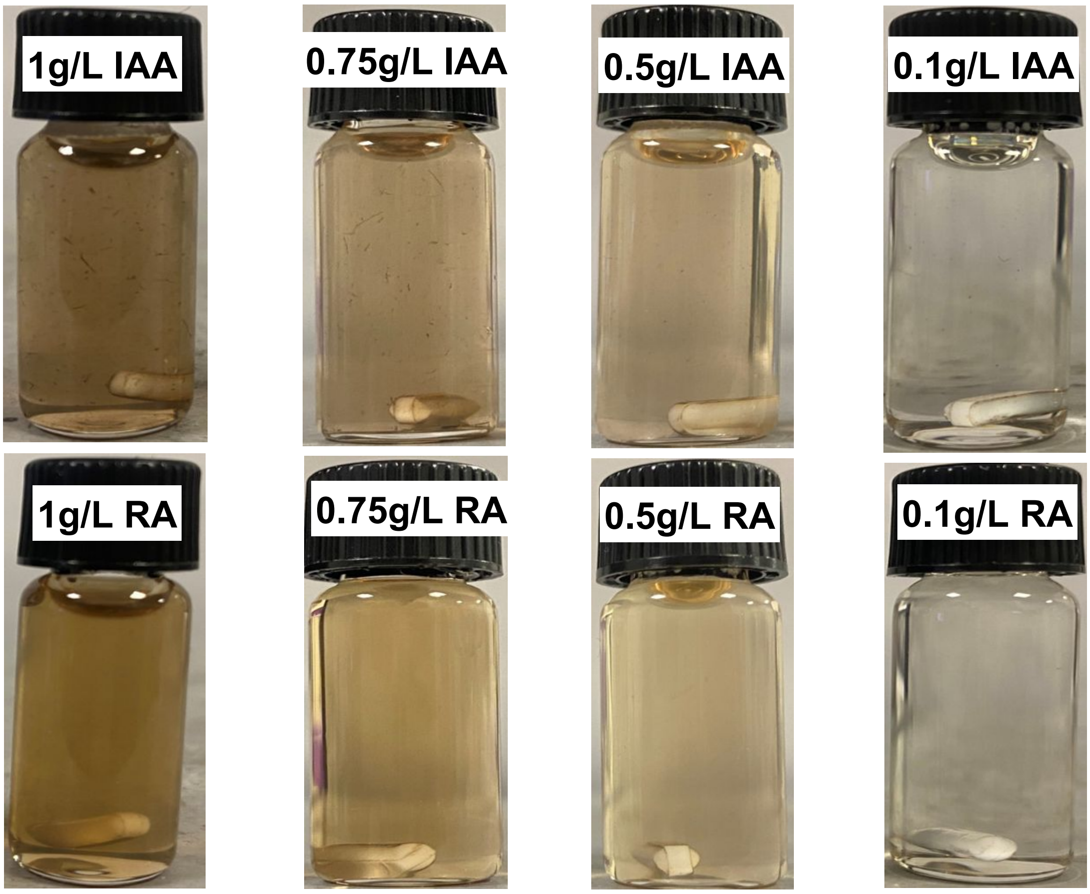

Figure S5. Images of waxy solutions in 6:4 DodecTol in Crystal 16 vials taken while being heated and agitated at 700rpm. The sedimented large IAA aggregates are visible.

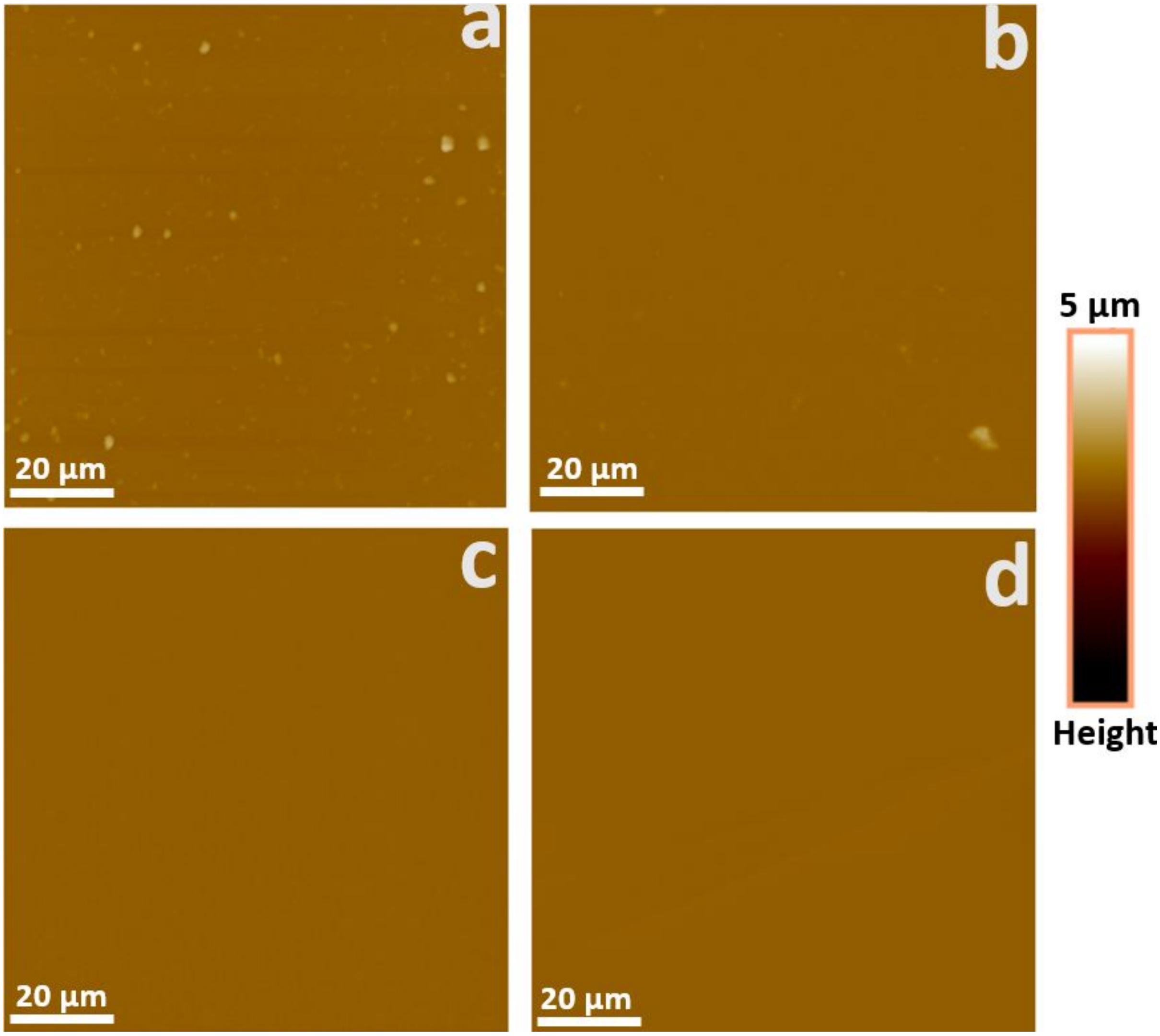

Figure S6. Raw AFM images of RA and IAA aggregation behavior and cluster sizes formed by fast drying solutions of 0.1 and 0.01g/L RA or IAA in toluene. (a) 0.1 g/L IAA, (b) 0.01 g/L IAA, (c) 0.1 g/L RA, and (d) 0.01 g/L RA.

S5. Polythermal plots of C_{28} at fixed 1000:1 wt/wt ratio in 6:4 DodecTol and Toluene

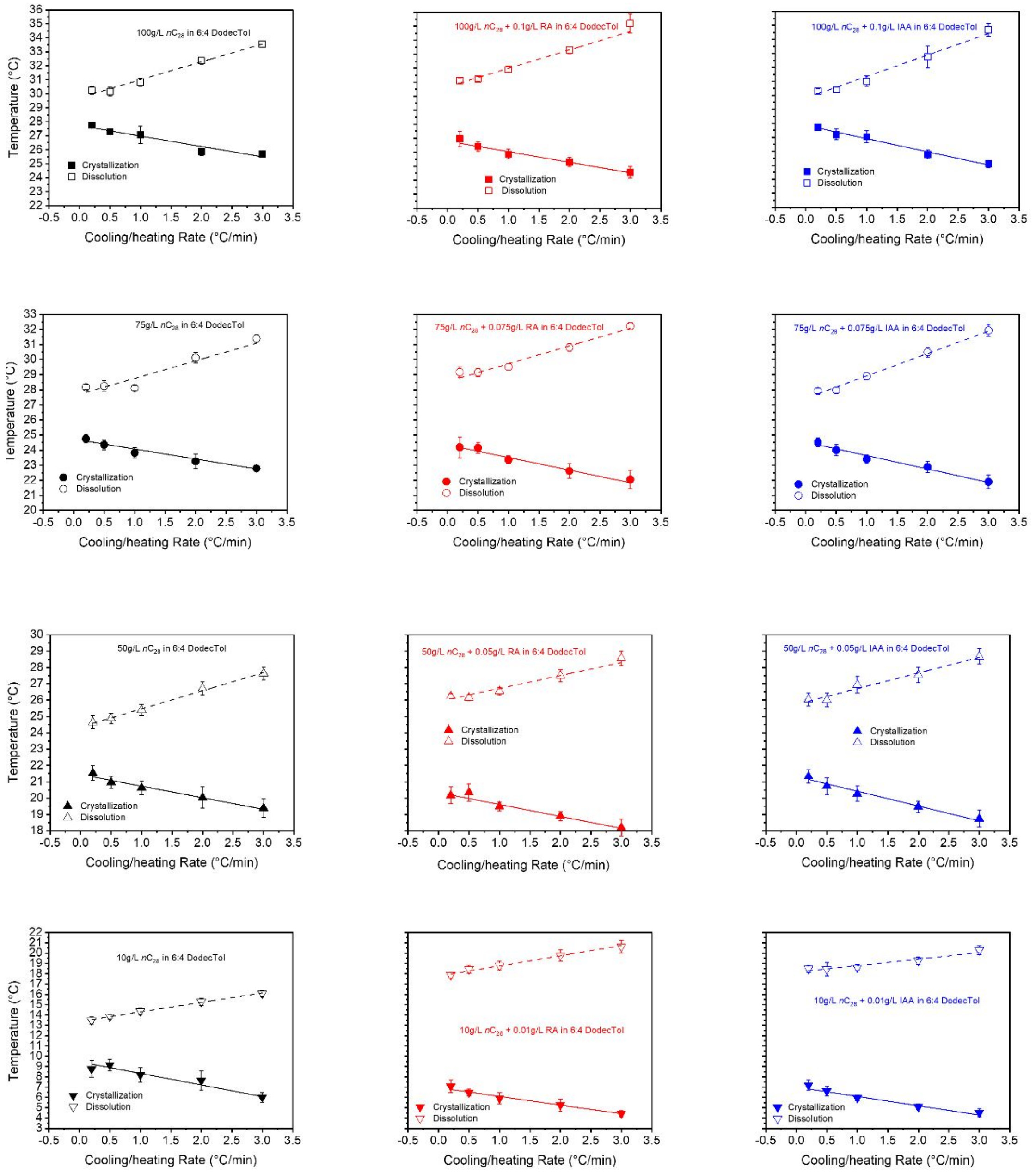

Figure S7. Plots of T_c and T_{diss} as a function of q, with the corresponding best linear fits for 100, 75, 50, 10 g/L C_{28} with and without 0.1g/L RA or IAA at 1000:1 wt/wt C_{28}: asphaltene ratio in 6:4 DodecTol solution.

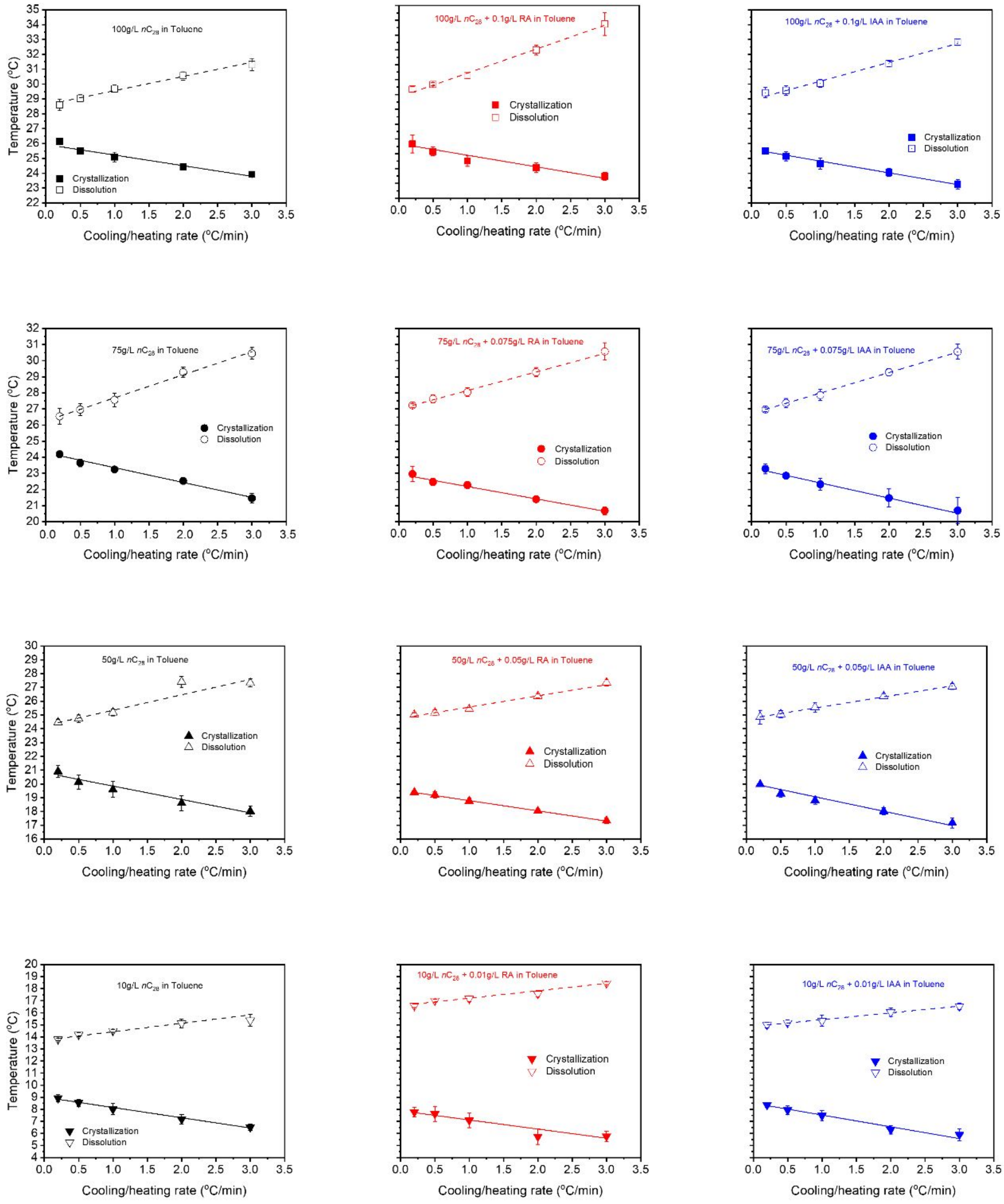

Figure S8. Plots of T_c and T_{diss} as a function of q, with the corresponding best linear fits for 100, 75, 50, 10 g/L C_{28} with and without 0.1g/L RA or IAA at 1000:1 wt/wt C_{28}: asphaltene ratio in 6:4 toluene solution.

S6. Nucleation Mechanism – *ln q vs ln u_c*

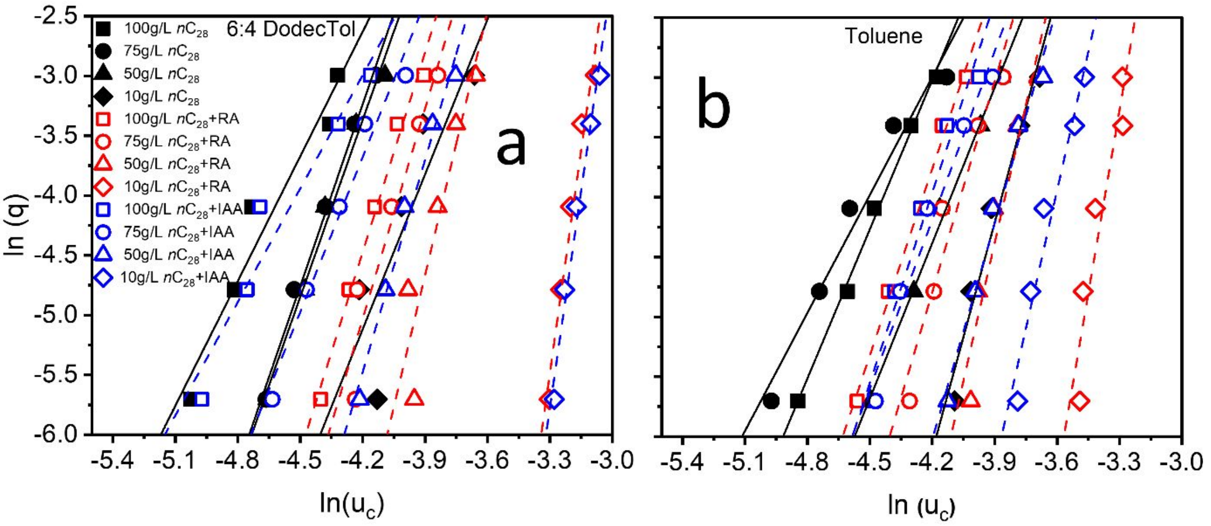

Figure S9. *ln q* vs *ln u_c* plots for used for determining the nucleation mechanisms of C_{28} at a concentration of 100, 75, 50, 10 g/L C_{28} with and without RA or IAA at 1000:1 wt/wt C_{28}: asphaltene ratio in (a) 6:4 DodecTol and (b) toluene.

S7. Nucleation kinetics for progressive nucleation (PN) – *ln q vs u_c*

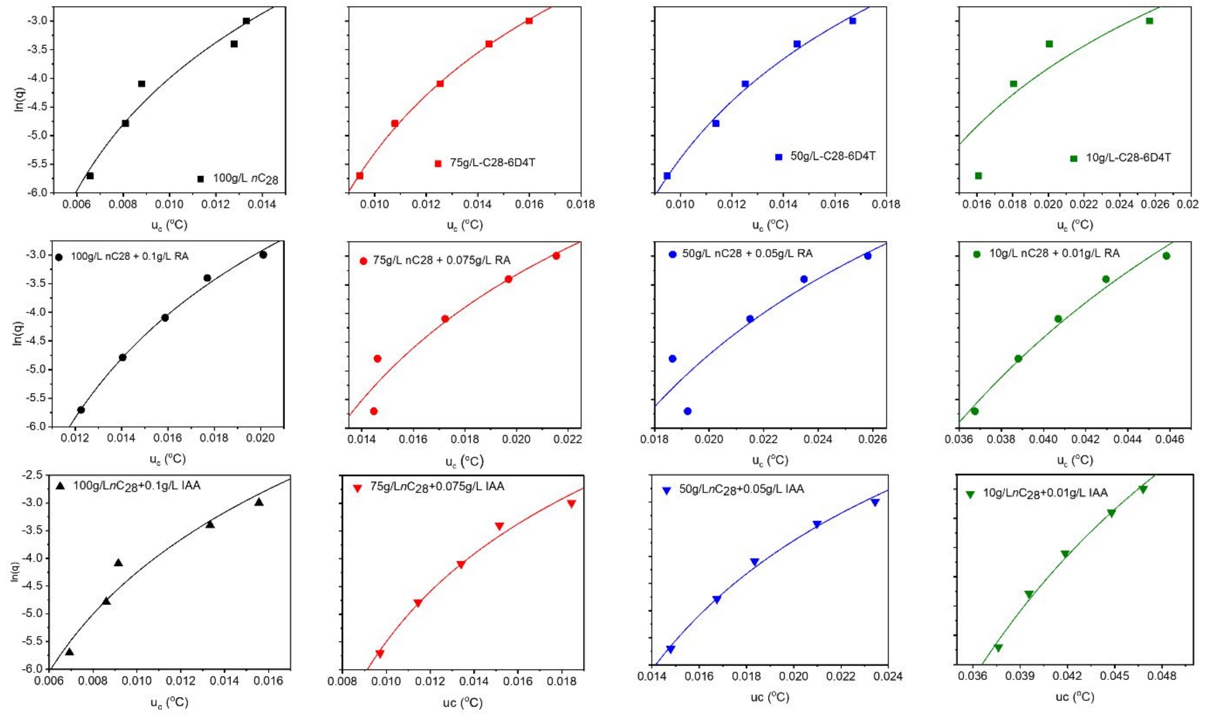

Figure S10. u_c vs *ln q* plots fitted with PN equation used to determine the nucleation kinetics parameters of 100, 75, 50, 10 g/L C_{28} with and without 0.1g/L RA or IAA at 1000:1 wt/wt C_{28}: asphaltene ratio in 6:4 DodecTol.

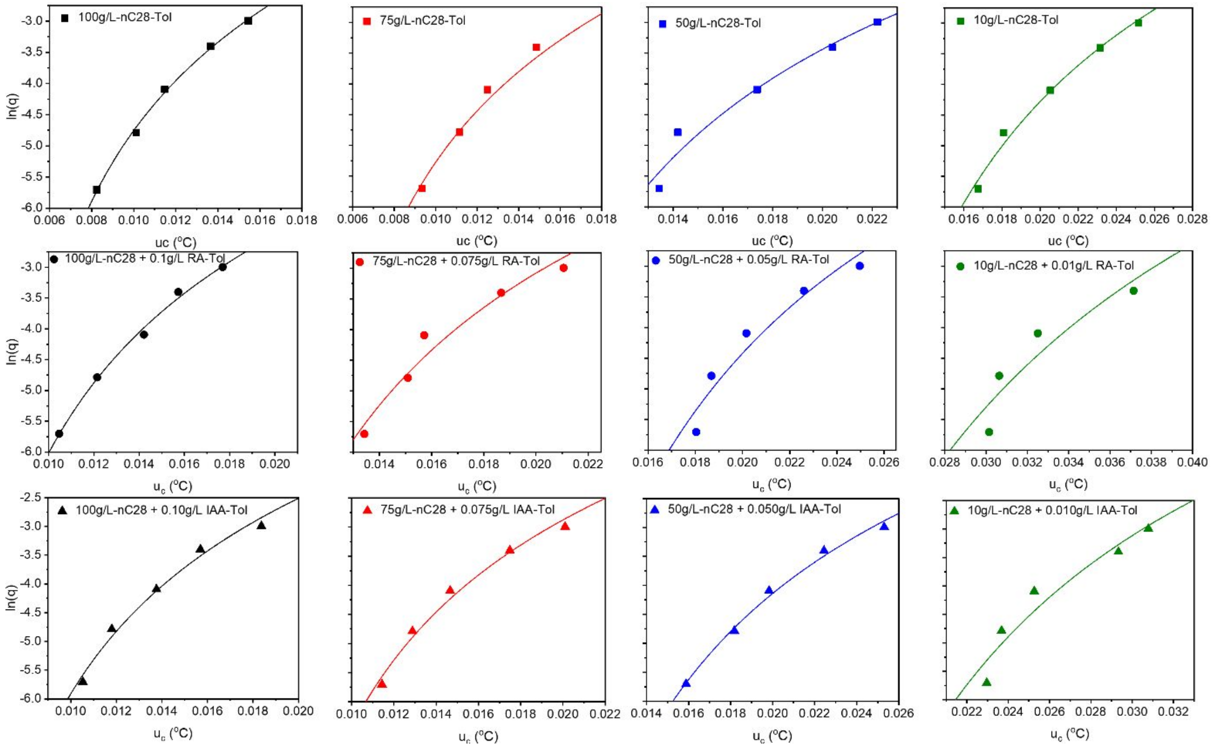

Figure S11. u_c vs *ln q* plots fitted with PN equation used to determine the nucleation kinetics parameters of 100, 75, 50, 10 g/L C_{28} with and without 0.1g/L RA or IAA at 1000:1 wt/wt C_{28}: asphaltene ratio in toluene.

Table S3. Nucleation mechanisms determined from the slope of u_c vs q in *ln*−*ln* coordinates.

Asp. Type	nC$_{28}$ concentration (g/L)	Slope (ω)	R^2	Nucleation mechanism	Slope (ω)	R^2	Nucleation mechanism
		**6:4 DodecTol**			**Toluene**		
No asphaltene	100	3.48	0.94	PN	4.16	0.99	PN
	75	5.06	0.99	PN	3.29	0.96	PN
	50	4.93	0.98	PN	4.33	0.99	PN
	10	4.32	0.73	PN	6.37	0.96	PN
RA	100	5.57	0.98	PN	5.20	0.99	PN
	75	5.86	0.91	PN	5.88	0.94	PN
	50	7.34	0.84	PN	7.68	0.91	PN
	10	12.40	0.97	PN	10.20	0.88	PN
IAA	100	3.16	0.93	PN	5.30	0.95	PN
	75	4.32	0.97	PN	4.70	0.97	PN
	50	5.92	0.98	PN	6.01	0.98	PN
	10	12.10	0.98	PN	7.71	0.94	PN

Table S4. Calculated nucleation kinetic parameters from KBHR Analysis obtained for C_{28} in 6:4 DodecTol with and without RA or IAA at 1000:1 wt/wt ratio. r^* and i^* are calculated at u_c corresponding to 3 °C/min.

C_{28} concentration (g/L)	6:4 DodecTol				
	b	γ_{eff} (mJ/m^2)	r^* (nm)	i^*	$i^* = r^*/r_{28}$
**C_{28}**					
10	2.41E-04	2.15	0.80	1.04	3.71
50	1.47E-04	1.85	1.05	2.40	4.90
75	1.50E-04	1.86	1.07	2.47	4.95
100	2.43E-05	1.02	0.73	0.80	3.40
**C_{28} + RA**					
10	7.80E-03	6.90	1.43	6.00	6.66
50	1.00E-03	3.50	1.30	4.40	6.00
75	4.27E-04	3.65	1.18	3.30	5.50
100	3.07E-04	2.40	1.12	3.00	5.30
**C_{28} + IAA**					
10	7.86E-03	6.91	1.41	5.70	6.55
50	4.98E-04	2.80	1.13	3.00	5.30
75	1.16E-04	1.71	0.90	1.41	4.20

100	1.48E-05	0.90	0.60	0.30	2.50

Table S5. Calculated nucleation kinetic parameters from KBHR Analysis obtained for C28 in toluene with and without RA or IAA at 1000:1 wt/wt ratio. r^* and i^* are calculated at u_c corresponding to 3 °C/min.

$n\mathrm{C}_{28}$ concentration (g/L)	**Toluene**				
	b	γ_{eff} (mJ/m^2)	r^* (nm)	i^*	$i^* = r^*/r_{28}$
			**C_{28}**		
10	6.94E-04	3.06	1.16	3.20	5.40
50	2.63E-04	2.25	1.02	2.20	4.75
75	9.37E-05	1.60	0.94	1.70	4.40
100	8.23E-05	1.55	0.95	1.75	4.42
			**C_{28} + RA**		
10	3.63E-03	5.35	1.36	5.10	6.32
50	1.06E-03	3.60	1.36	5.15	6.33
75	4.12E-04	2.60	1.18	3.35	5.50
100	1.93E-04	2.05	1.10	2.70	5.10
			**C_{28} + IAA**		
10	1.75E-03	4.20	1.30	4.20	5.92
50	5.77E-04	2.90	1.10	2.60	5.10
75	1.96E-04	2.05	0.97	1.85	4.50
100	1.75E-04	2.00	1.00	2.10	4.63

References

(1) Wang, X.; Wei, Y.; et al. Phase behaviors of n-octacosane in nanopores: Role of pore size and morphology. *Thermochimica Acta* **2020**, *690*, 178687. DOI: https://doi.org/10.1016/j.tca.2020.178687.
